# Phosphorus availability modifies the responses of Glomeromycotina and Mucoromycotina to nitrogen addition

**DOI:** 10.1007/s00572-026-01256-5

**Published:** 2026-03-26

**Authors:** Jiří Košnar, Marie Šmilauerová, Tereza Pecková, Petr Šmilauer

**Affiliations:** Faculty of Science, University of South Bohemia in České Budějovice, Branišovská 1760, České Budějovice, CZ-370 05 Czech Republic

**Keywords:** Arbuscular mycorrhizal fungi (AMF), Diversity, Fine root endophytes (FRE), Fertilisation, Grassland, Nitrogen, Phosphorus

## Abstract

Grassland fertilisation modifies the relationship between mycorrhizal fungi and their plant hosts. The composition and diversity of the fungal community depend on the background nutrient availability and changes in nutrient availability caused by fertilisation. We determined the effects of nitrogen and phosphorus addition at 12 sites with different background nutrient availabilities using 5 species of bait seedlings planted in the field. We studied two groups of mycorrhizal fungi – arbuscular mycorrhizal fungi (Glomeromycotina, G-AMF) and fine root endophytes (Mucoromycotina, M-FRE) – using molecular methods and light microscopy. Both fungal communities showed large differences among forb and grass host species and among individual sites. G-AMF community composition varied more along background nutrient availability gradients than with short-term fertilisation. Phosphorus availability affected the composition of both fungal communities, but nitrogen availability only affected G-AMF community composition. Nitrogen fertilisation affected both fungal communities more than phosphorus fertilisation, and the effects of nitrogen addition varied with the local phosphorus availability. Our study provides an understanding of the long-term adaptation of mycorrhizal symbiosis to nutrient availability and how it interacts with nutrient addition.

**Supplementary Information:**

The online version contains supplementary material available at 10.1007/s00572-026-01256-5.

## Introduction

Of vascular plant species, 92% form a mycorrhizal symbiosis with several groups of fungi (Brundrett and Tedersoo 2018). In grasslands, two groups assigned to the Mucoromycota phylum dominate mycorrhizal symbiosis - arbuscular mycorrhizal fungi from the Glomeromycotina subphylum (G-AMF) and fine root endophytes from the Mucoromycotina subphylum (M-FRE), which were neglected until recently (Albornoz et al. 2021).

G-AMF and M-FRE provide plant hosts with phosphorus (P) and nitrogen (N) in exchange for assimilated carbon (C) (Field et al. 2019; Bennet and Groten 2022), and G-AMF are known to provide other services, such as pathogen resistance and enhanced water flow (Delavaux et al. 2017). The hyphae of fungal symbionts grow in two environments simultaneously – in host roots and in the surrounding soil space (Powell and Rillig 2018). To accomplish their symbiotic roles, G-AMF from different phylogenetic groups have evolved variations in their relative C investment into intra- and extra-radical hyphal parts (Powell et al. 2009). Together with the presumed differences in provided services, this variation is the basis for assigning G-AMF families into functional guilds, as proposed by Chagnon et al. (2013) and formalised by Weber et al. (2019).

Although plants obtain a substantial portion of N and P through mycorrhizal fungi, the fungal symbiont cannot be considered a mere agent of nutrient acquisition by plants. Symbiotic fungi require the same macronutrients and therefore compete with plants, particularly for N (Boussageon et al. 2022), which is present in a higher proportion in the fungal biomass compared to plants (Johnson 2010). In our previous study (Košnar et al. 2025a) we observed significant effects of N addition on G-AMF community composition and α-diversity, but not when we added only P.

The strength and nature of mycorrhizal symbiosis are modified by the natural variation in nutrient availability and by the fertilisation of agricultural land, which provides additional N, often combined with P and potassium. Additional N input reduces G-AMF biomass (Camenzind et al. 2014; Han et al. 2020; Ma et al. 2021) and community richness (Ceulemans et al. 2019; Ma et al. 2021). Increased N availability reduces root colonisation and the taxonomic richness of M-FRE (Kowal et al. 2022; Seeliger et al. 2024). The richness of G-AMF declines as P availability increases (Camenzind et al. 2014; Ceulemans et al. 2019; Hu et al. 2019; Frew et al. 2023). A similar response to P availability has also been observed for M-FRE in temperate Australia (Albornoz et al. 2021). The reduced abundance or diversity of fungal symbionts due to excessive fertilisation might affect ecosystem functioning (Ma et al. 2021).

Nutrient addition also affects mycorrhizal symbiosis by increasing the plant aboveground biomass. The consequent shading limits the incoming light for less competitive plants (Neuenkamp et al. 2021), including seedlings. This shading limits the ability of smaller plants to trade C for N and P (Knegt et al. 2016) with fungal symbionts. With fertilisation, soil nutrients become more available for direct uptake by plant roots and the host can limit the C supply to non-essential symbionts (Johnson 2010). When adding N as nitrates, fungal communities may be affected by soil acidification (Chen et al. 2018; Hu et al. 2019; Davison et al. 2021). The indirect effects of light limitation and acidification provide an argument for examining the effects of fertilisation on fungal communities under field conditions.

The responses of individual mycorrhizal fungal taxa to fertilisation depend on their life strategy, including their efficiency in nutrient acquisition for their plant hosts. Although little is known about the effects of N and/or P fertilisation on individual M-FRE taxa, previous studies regarding G-AMF have shown that fertilisation particularly affects fungal taxa with efficient P uptake, namely those in the Gigasporaceae and Diversisporaceae families from the edaphophilic guild (Weber et al. 2019). These fungal taxa develop extensive extra-radical hyphal systems that demand a large amount of C for growth and maintenance and are therefore most affected by C limitation (Powell et al. 2009). Fungal taxa from the rhizophilic guild (including the Glomeraceae and Claroideoglomeraceae families) persist better under fertilised conditions (Frew et al. 2023), likely due to their limited hyphal system (Powell et al. 2009). Johnson (2010) suggested that the abundance and diversity of symbiotic fungi increased after N fertilisation at sites with low P availability, a pattern that has been demonstrated in other studies (Treseder et al. 2018; Han et al. 2020; Ma et al. 2021). To better understand the response of fungal symbionts to fertilisation, the consistency of the response of G-AMF functional guilds to nutrient addition needs to be determined, and data on the fertilisation-related responses of individual M-FRE operational taxonomic units (OTUs) should be collected.

G-AMF co-adapt with plant hosts to match the local long-term nutrient availability at particular sites (Johnson et al. 2010). Such co-adaptation and selection of fungal symbionts is likely driven by the ability of a plant to “pay” more of the C reward to fungal symbionts providing a higher amount of P (Bever 2015). Boerner (1990) found that G-AMF from sites with a low P availability provided plants with more P than fungi from P-rich sites. In contrast, Lekberg et al. (2024) found an opposite pattern and a different G-AMF community composition at sites with high P availability. Host plants inoculated with G-AMF from their home site have greater variation in productivity, and the host response varies with the identity of fungal symbionts (Klironomos 2003). Adaptation of the mycorrhizal relationship to nutrient availability likely occurs through changes in the fungal community composition and the adaptations of present fungal taxa (Antunes et al. 2012). When determining the effects of fertilisation on mycorrhizal fungi, it is important to compare fertilisation effects with the variation in the fungal community along long-term nutrient availability gradients.

The efficiency of mycorrhizal symbiosis in nutrient exchange depends on fungal partners and host plant identity, particularly its functional group. Walder et al. (2012) found that a grass host supplied more C to G-AMF partners than co-cultivated forbs to gain a smaller share of N and P from the fungi than the forb. Similarly, Lekberg et al. (2024) found that a forb host species exchanged relatively smaller amount of C with G-AMF than grass hosts and was able to better regulate the P-to-C exchange ratio under increased P availability. In previous greenhouse study (Šmilauer et al. 2021b), we observed different responses of G-AMF communities to elevated nutrients between forb and grass hosts. Therefore, when determining the effects of nutrient availability on mycorrhizal symbiosis in a plant community, it is important to compare such effects among plant functional groups.

When designing field-based fertilisation experiment, the choice of age and mycorrhizal status of examined host plants are important practical considerations. At the time a symbiotic relationship is established, a host plant (e.g., a newly emerged seedling) might support a wider range of G-AMF colonisers (Bever 2015). Some G-AMF taxa have therefore developed a ruderal strategy that involves speedy colonisation with limited persistence in the roots (Sýkorová et al. 2007). The use of bait seedlings, exposed to field conditions with no symbionts initially present in their roots, could reveal a wider spectrum of fungal taxa compared to adult plants grown under field conditions (Šmilauer et al. 2021a).

We examined the relative importance of background nutrient availability and fertilisation on the composition and richness of symbiotic fungal communities and compared these effects across two important fungal symbiont groups. Our study explored the response of G-AMF and M-FRE communities to nutrient addition (factorially combined inorganic N and P) across grassland sites, which differed in the background availability of N and P. We used bait seedlings from five species planted in plots with different fertilisation treatments and tested four hypotheses concerning community composition, taxonomic diversity, root colonisation rate, and relative abundance of G-AMF and M-FRE. We investigated the following hypotheses:

### H1:

Fungal communities in the roots of forbs respond more strongly to nutrient addition than those in grass roots, as forbs are better able to regulate the C-to-nutrient exchange ratio after a change in nutrient availability.

### H2:

N addition affects the fungal community composition and reduces taxonomic diversity more than P addition, as the N supply is more limiting for symbiotic partners than P. The response of the fungal community to N or P addition also differs between G-AMF and M-FRE groups because N limitation is less severe for M-FRE, as they can utilise organic N resources.

### H3:

The background (longer-term) availability of N or P at a site affects G-AMF and M-FRE communities more strongly than the short-term effects of N or P fertilisation because the background availability is reflected in the fungal community composition due to the co-adaptation process.

### H4:

The effects of fertilisation on symbiotic fungal communities differ depending on the background nutrient availability. Specifically, the effects of N addition on G-AMF and M-FRE communities depend on the background availability of P, based on the pattern described by Johnson (2010).

## Materials and methods

### Study sites and experimental design

This study used data collected from 12 sites located near České Budějovice, Czech Republic. These sites represent mown mesic grasslands with varying N and P availabilities: inorganic N 4.10–23.96 (median 9.13) mg.kg^− 1^ of dry soil; inorganic P 5.57–60.42 (median 30.57) mg.kg^− 1^ of dry soil. We established eight experimental 1 × 1 m plots at each site, with 2 replicates of the 4 treatments: control – with no nutrients added; N – with inorganic N added; P – with inorganic P added; and N + P – with both inorganic N and P added. The plots were fertilised in April and September 2022 and March 2023 (2 L of water containing 7.6 g K_2_HPO_4_ for P and N + P plots and/or 20 g NH_4_NO_3_ for N and N + P plots; this corresponds to 27 kg.ha^− 1^.y^− 1^ of P and 138 kg.ha^− 1^.y^− 1^ of N in year 2022 and half of that amount in the next year). Seedlings of five species (two forb species *Plantago lanceolata* and *Centaurea jacea*, and two grass species *Anthoxanthum odoratum* and *Poa angustifolia*, at each of 12 sites, and another forb, *Betonica officinalis*, only at four sites) were planted at experimental sites in spring 2023, with 12 replicates of each species per plot. Seedlings were planted into six groups within each plot (each group with two replicates of planted species), forming in each group a ring around a central colour plastic stick. Each seedling was planted within a small rubber ring (inner diameter 1 cm), fixed to soil surface with a split pin stuck into the ground. In addition to whole plot fertilisation, we also fertilised the seedling at days 10 and 38 after planting, with 80 mL of water per seedling group containing dissolved 0.52 g of K_2_HPO_4_ in P and N + P plots, and 1.35 g of NH_4_NO_3_ in N and N + P plots. Seedlings were collected after 11 weeks of field exposure. The sites and experimental design are described in detail in Appendix EA1.

### Sampling and processing of field samples

We washed the roots from collected seedlings, pooled the roots of the same plant species within each plot and used them to characterise the G-AMF and M-FRE communities (quantified using different primer pairs) and estimate the intensity of root colonisation by both groups under a microscope (BX50, Olympus) using 200× magnification, after staining with Chlorazol Black E (when the amount of available roots was sufficient). The aboveground parts of the seedlings were dried and, after pooling them across the plots with identical fertilisation treatments in the same or nearby sites to obtain the required dry weight, the N and P contents were assessed.

To estimate the background nutrient availability, we sampled plant community in the experimental plots from an 0.5 × 0.5 m area in summer 2024 (as plot-level fertilisation continued into spring 2024) to determine the N and P contents in the aboveground biomass. We collected the biomass in the third year of fertilisation to assure that the biomass on fertilised plots had a saturated content of added nutrients. Using an approach similar to Ostertag and DiManno (2016), we calculated the ratio of the N concentration in control plots to the concentration in N-fertilised plots to estimate the background availability of N at each site. We used a corresponding approach for P availability. The background availabilities represent the fractional saturation of community biomass with a particular nutrient, ignoring among-species differences in the nutrient concentration, as appropriate for a site characteristic. Soil was sampled at the same time to measure the available nutrients and the pH of individual plots to assess fertilisation effects.

Sampling and processing of the collected material (including final sample counts per species and treatment) are described in detail in Appendix EA1.

### DNA extraction, amplification and sequencing

We extracted DNA from seedling roots by following the cetyltrimethylammonium bromide (CTAB) protocol (Doyle and Doyle 1987). Then, we purified DNA by using the PowerClean Pro DNA Clean-Up Kit (Qiagen, Hilden, Germany). DNA extracts with a high DNA concentration were diluted in sterile water (1:10 v/v).

For amplicon sequencing of the G-AMF communities, we used a standard semi-nested polymerase chain reaction (PCR) to amplify the 550-base pair (bp) amplicon of small subunit ribosomal DNA (SSU rDNA) following Dumbrell et al. (2011), except we used the AML2 primer (Lee et al. 2008) as a AMF-specific primer, in pair with the NS31 primer. The second PCR used the WANDA primer instead of the NS31 primer, fused to sample-specific barcodes. The amplicons were sequenced (paired-end 2 × 300 bp) with Illumina equipment at the Institute of Applied Biotechnologies (Prague, Czech Republic).

To analyse the M-FRE community (and to compare the relative frequencies of G-AMF and M-FRE DNA sequences), we sequenced 280-bp PCR amplicons of SSU rDNA using the AMV4.5NF and AMDGR primers (Sato et al. 2005). Both primers were fused with sample-specific barcodes and sequenced (paired-end 2 × 250 bp) with Illumina equipment by the SEQme (Dobříš, Czech Republic).

Appendix EA2 provides additional details about the DNA analysis protocols.

### Bioinformatic analysis

We processed the next-generation sequencing (NGS) data with the software tools in SEED ver. 2.0 (Větrovský et al. 2018), mothur ver. 1.39.5 (Schloss et al. 2009) and PipeCraft ver. 1.0 (Anslan et al. 2017). We used FLASH ver. 1.2.11 (Magoč and Salzberg 2011) to assemble paired-end reads, VSEARCH ver. 1.11.1 (Rognes et al. 2016) for quality filtering, and SEED ver. 2.0 for demultiplexing. We identified the final G-AMF sequences by using the ssu pipeline (Vasar et al. 2017) with the extended MaarjAM database (Öpik et al. 2010) and the final M-FRE sequences with locally built Endogonales database based on the SILVA database (Quast et al. 2013). The relative proportion of G-AMF and M-FRE DNA sequences (both originating from the sequencing based on AMV4.5NF – AMDGR primer pair) was estimated as G-AMF/M-FRE sequence ratio for individual samples. Appendix EA3 includes additional details about pre-processing the raw NGS reads, identification of G-AMF virtual taxa (VTXs) and M-FRE OTUs, and estimation of the G-AMF/M-FRE sequence ratio.

### Statistical analysis

Using the R software, ver. 4.4.1 (R Core Team 2024), we estimated linear mixed-effect models (LMMs) to summarise the effects of various predictors on the colonisation of roots by G-AMF and M-FRE, their α-diversity (Hill’s N2 index, Legendre and Legendre 2012) and taxonomic richness, and the G-AMF/M-FRE ratio. We analysed the variation in community composition (i.e. β-diversity) using multivariate constrained ordination with the Canoco software, ver. 5.15 (ter Braak and Šmilauer 2018). Appendix EA4 provides details of the fitted statistical models.

## Results

We identified 107 VTXs (belonging to seven families) from the Glomeromycotina subphylum and 19 OTUs from the Mucoromycotina subphylum in 327 and 356 samples of seedling roots, respectively. Appendix EA5 provides additional details.

### Fertilisation effects on seedlings, soil and plant community biomass

In the aboveground biomass of the experimental seedlings, the N and P contents increased with N or P fertilisation (N: from 2.17% in control plots to 4.77% in N plots and 4.28% in N + P plots; P: from 0.35% in control plots to 0.59% in P plots and 0.71% in N + P plots), and the N/P ratio changed significantly (from 6.6 in control plots) when N or P were applied separately (to 12.8 in N plots and 3.7 in P plots).

Consistent responses to applied nutrients were also confirmed by analyses of soil chemistry and N and P contents in the aboveground biomass of the surrounding community. Further information can be found in Appendix EA6, including Tables EA4 – EA9.

### Effects of among-site variation and plant hosts on fungal symbionts

G-AMF and M-FRE community composition varied among sites and host species, mainly between forbs and grasses (Table 1). The N2 diversity of the G-AMF community did not differ among host species and only weakly among sites, whereas its taxonomic richness did not vary among sites and only weakly among host species. We found no variation in M-FRE diversity among host species or sites, but there was a stronger and significant variation in M-FRE taxonomic richness (Table 1). There were significant differences in the G-AMF/M-FRE ratio among host species and sites (Table 1).

Root colonisation by G-AMF and M-FRE varied strongly and significantly among sites and particularly among host species. For G-AMF colonisation was higher in forb species (61.9–81.2%) than in grass species (30.0–52.7%), For M-FRE symbionts, colonisation did not differ between the two host functional groups. The lowest M-FRE colonisation (5.9%) was observed in grass species *Poa angustifolia*, and the highest colonisation (42.0%) was in forb species *Plantago lanceolata*.

**Table 1:** Summary of the statistical models examining the effects of host species and site identity on the composition, α-diversity (Hill’s N2 index), taxonomic richness, DNA sequence ratio, and colonisation level of the G-AMF and M-FRE communities, using partial RDA (for compositional variation) and LMM (for other community attributes). The test statistic is pseudo-F (without DFs) for RDA and the likelihood-ratio statistic (χ^2^) for LMM, with DF = 4 for the host species effect. Among-site variation was tested using the likelihood-ratio test of a random effect (DF = 1). G – grasses (AO – *Anthoxanthum odoratum*, PA – *Poa angustifolia*), F – forbs (BO – *Betonica officinalis*, CJ – *Centaurea jacea*, PL – *Plantago lanceolata*); *n.s.* – not significant, *n.a.* – not appropriate. Significance estimates with 0.05 < *p* < 0.10 are shown in parenthesis and interpreted as a non-significant test outcome

	Host species effect			Among-site variation	
	Test statistic / *p*-value	Explained variation [%]	Effect description	Test statistic / *p*-value	Explained variation [%]
Variation of G-AMF community	F = 5.9 *p* < 0.001	4.8%	F separated from G on the first axis	F = 4.2 *p* < 0.001	9.1%
Variation of M-FRE community	F = 2.5 *p* = 0.003	1.2%	F separated from G on the first axis	F = 3.7 *p* < 0.001	7.5%
α-diversity of G-AMF community	χ^2^ = 5.8 n. s.	1.6%	n. a.	χ^2^ = 3.2 *p* = 0.037	6.0%
Richness of G-AMF community	χ^2^ = 9.5 *p* = 0.050	2.8%	PA > AO *p* = 0.046	χ^2^ = 1.8 n.s.(*p* = 0.088)	5.3%
α-diversity of M-FRE community	χ^2^ = 9.2 n.s.(*p* = 0.056)	2.5%	n. a.	χ^2^ = 2.0 n.s.(*p* = 0.078)	4.9%
Richness of M-FRE community	χ^2^ = 16.8 *p* = 0.002	4.3%	BO > CJ & PA & AO	χ^2^ = 11.6 *p* < 0.001	9.8%
G-AMF/M-FRE ratio	χ^2^ = 23.6 *p* < 0.001	5.7%	PL & CJ & PA > AO	χ^2^ = 4.9 *p* = 0.014	7.8%
G-AMF root colonisation	χ^2^ = 85.0 *p* < 0.001	34.3%	F > G ^A^	χ^2^ = 7.0 *p* = 0.004	9.7%
M-FRE root colonisation	χ^2^ = 122.6 *p* < 0.001	40.4%	all species differ except CJ × AO ^B^	χ^2^ = 11.5 *p* < 0.001	13.7%

^A^ – BO had not its root colonisation extent significantly different from AO (which had lower colonisation level) or from CJ or PL; (PL & CJ) > AO > PA; PA colonisation was just 48% of the AO level

^B^ – PL > (AO & CJ) > BO > PA

### Effects of fertilisation on fungal symbionts

**Fig. 1:**
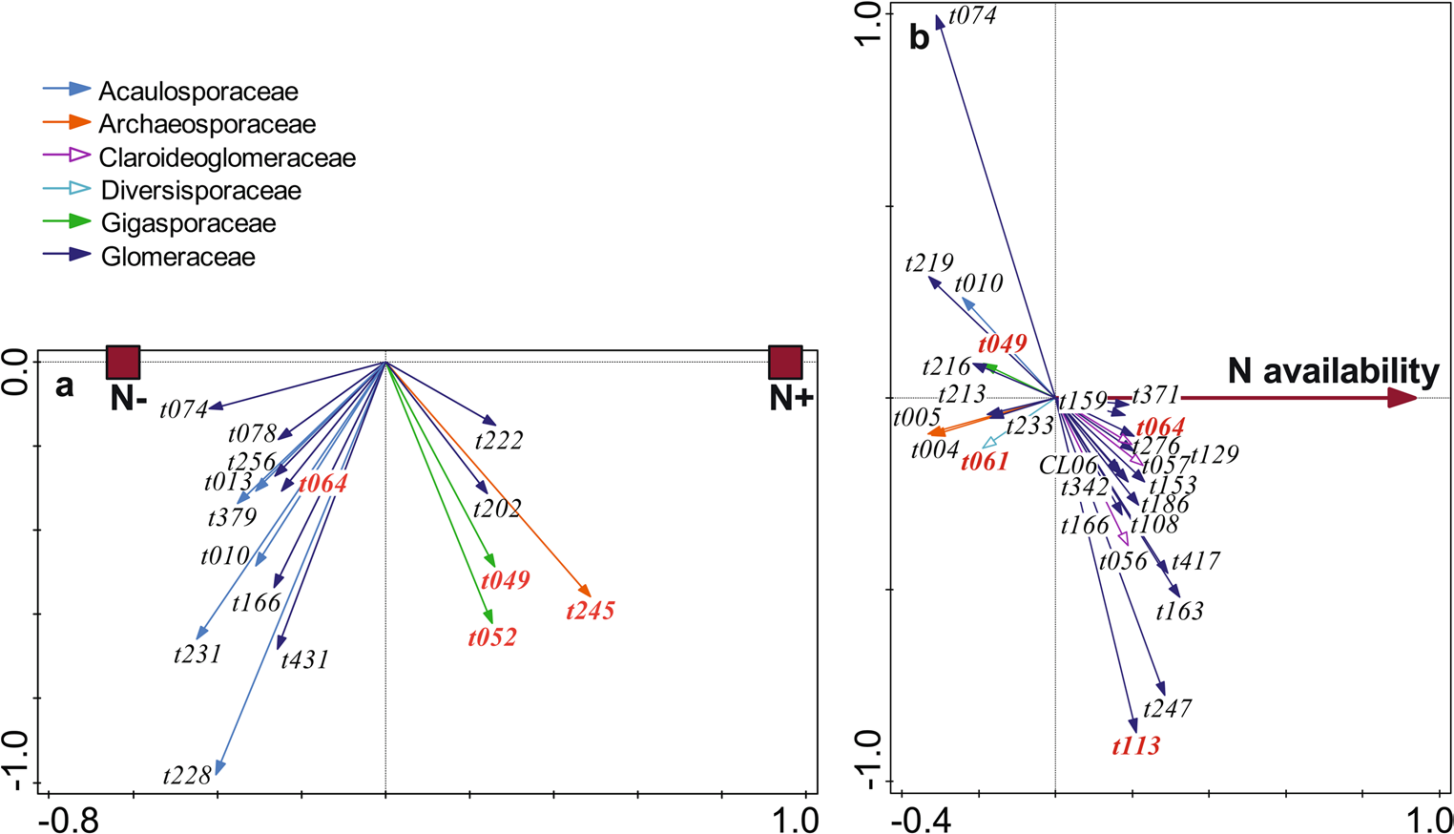
The effects of N addition (**a**, N + vs. N-) and background N availability (**b**) on G-AMF community composition, shown as biplots of partial RDA. Only G-AMF taxa strongly affected by the predictors (based on t-value biplots) are shown, representing 33.6% (**a**) and 63.2% (**b**) of analysed DNA sequences. The arrow attributes show the assignment of VTXs to G-AMF families (the key is in the upper left corner). VTXs with red bold labels correspond to morphotaxa known from cultivation. For additional details about interpreting ordination biplots, see Appendix EA4

**Fig. 2:**
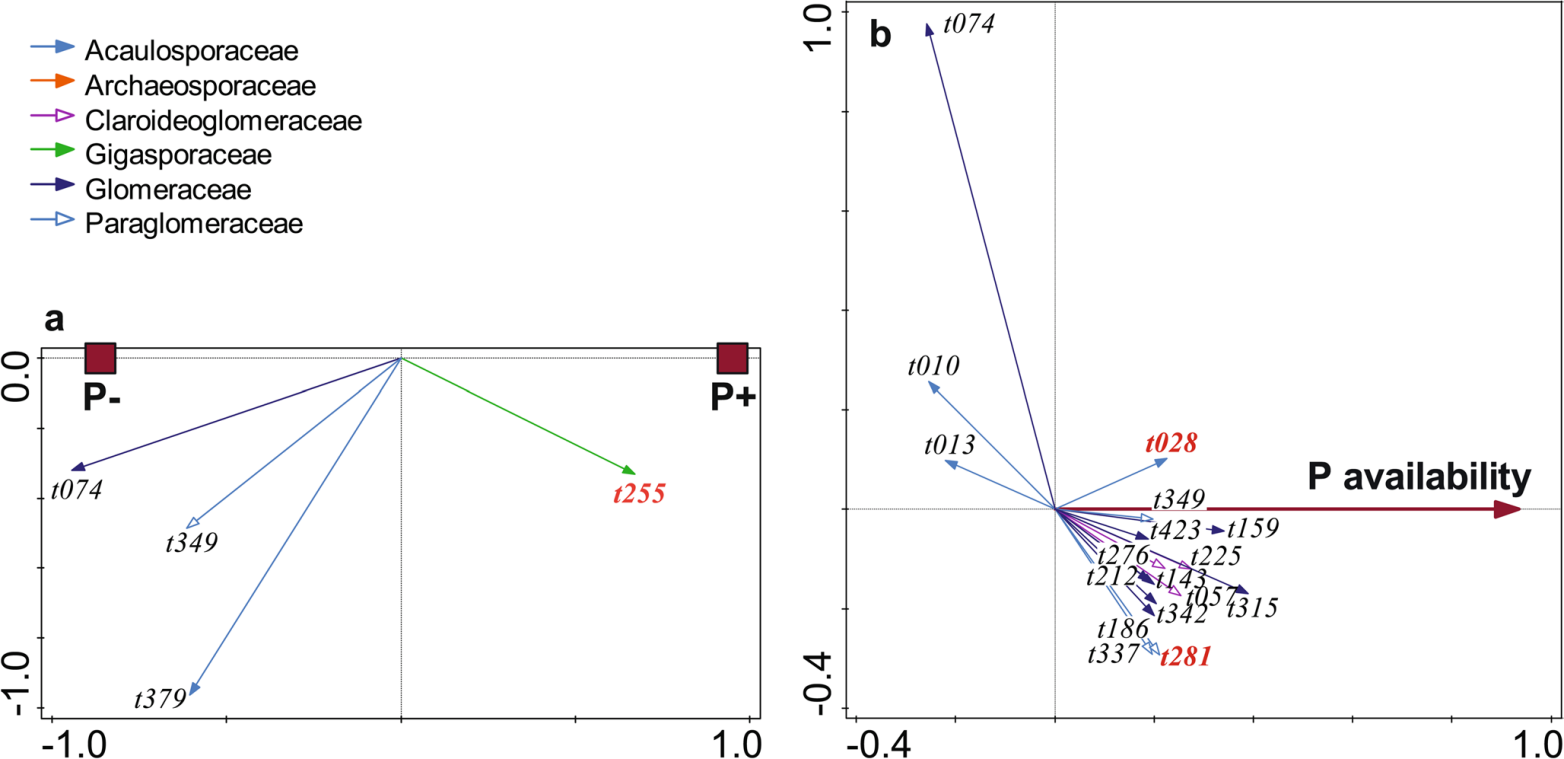
The effects of P addition (**a**, P + vs. P-) and background P availability (**b**) on G-AMF community composition, shown as biplots of partial RDA. Only the G-AMF taxa strongly affected by predictors (based on t-value biplots) are shown, representing 19.5% (**a**) and 28.3% (**b**) of analysed DNA sequences. The arrow attributes show the assignment of VTXs to G-AMF families (the key is in the upper left corner). VTXs with red bold labels correspond to morphotaxa known from cultivation. For additional details about interpreting ordination biplots, see Appendix EA4

**Fig. 3:**
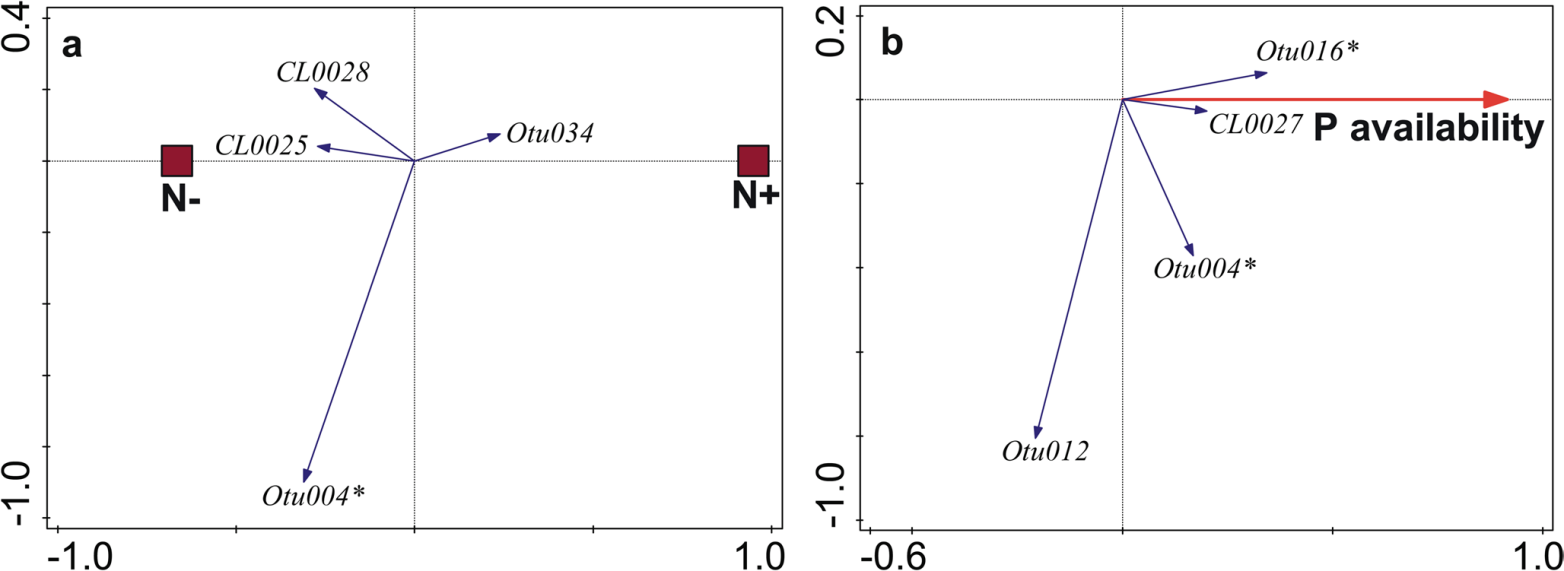
The effects of N addition (**a**, N + vs. N-) and background P availability (**b**) on M-FRE community composition, shown as biplots of partial RDA. Only the M-FRE OTUs strongly affected by predictors (based on t-value biplots) are shown, representing 63.4% (**a**) and 76.9% (**b**) of analysed DNA sequences. OTU labels ending with * refer to taxa already known as plant symbionts. For additional details about interpreting ordination biplots, see Appendix EA4

**Fig. 4:**
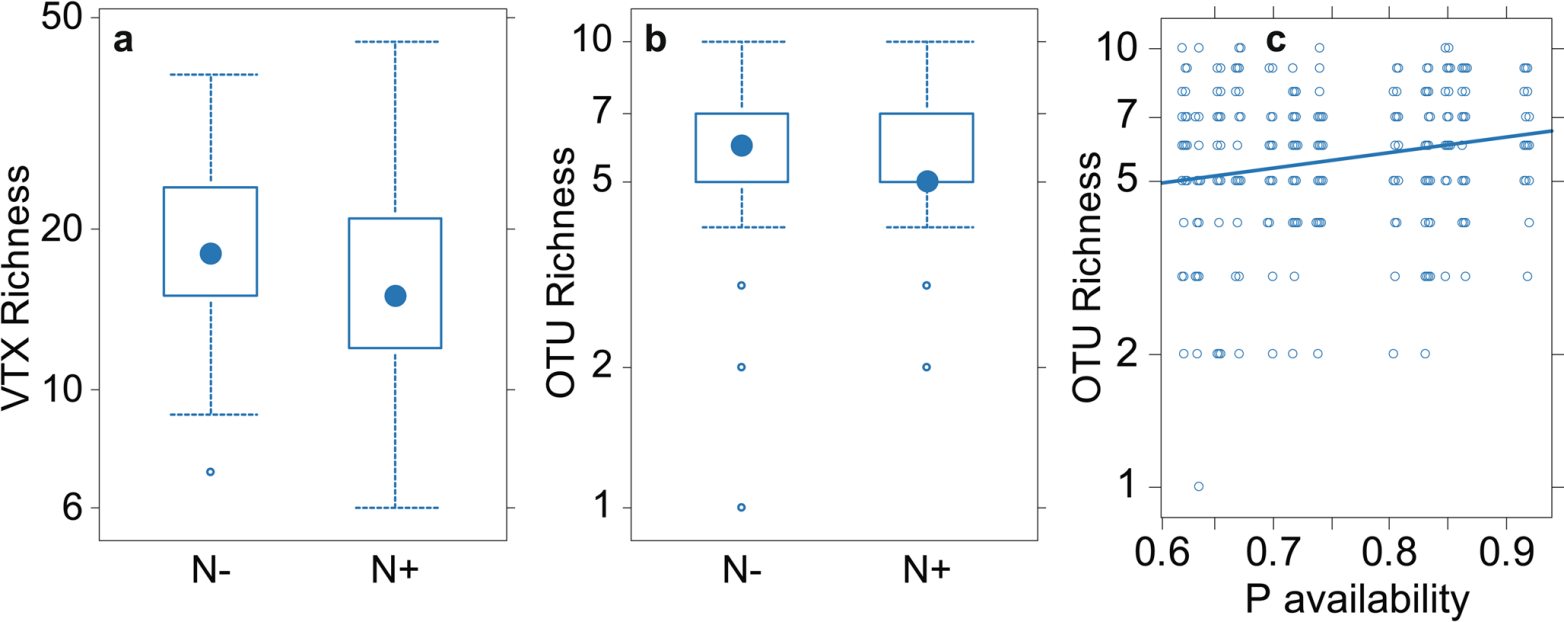
The effects of N addition on the richness of G-AMF VTXs (**a**) and M-FRE OTUs (**b**) and the effect of P background availability on M-FRE OTU richness (**c**). The box-and-whisker plots show the median richness as a blue filled circle within a box spanning from the lower to upper quartiles. The vertical axes use a log–scale, in accordance with the statistical models. The differences between N- and N+ treatments and the effect of P availability are significant at *p* = 0.022, *p* = 0.007, and *p* = 0.007, respectively for subplots **a**, **b**, and **c** (Tables 2 and 3)

G-AMF community composition responded significantly to N (Fig. 1a) and P addition (Fig. 2a), but compositional changes were weak (Table 2). M-FRE community composition showed a significantly strong response to N addition (Fig. 3a) compared to the G-AMF community, but P addition had no effect on the M-FRE community (Table 2). In the G-AMF community, N addition led to a significant 18% decrease in α-diversity and a 10% decrease in VTX richness (Fig. 4a). For the M-FRE community, N addition only affected OTU richness, with a significant 10% decrease (Fig. 4b; Table 2). There were no significant responses in diversity or richness due to P addition for either G-AMF or M-FRE. The colonisation of seedling roots by either G-AMF or M-FRE fungal symbionts was not affected by N or P addition, but N addition significantly decreased the G-AMF/M-FRE ratio by 42% (Table 2). This change was caused by a significant increase of the M-FRE DNA sequence counts (χ^2^_1_ = 8.03, *p* = 0.005) rather than by a drop in the count of G-AMF sequences (χ^2^_1_ = 0.99, n.s.). The interaction between the N and P effects was not significant for any of the attributes of the G-AMF or M-FRE community (Table 2).

**Table 2:** Summary of the statistical models examining the effects of N addition, P addition, and their interaction on the composition, α-diversity (Hill’s N2 index), taxonomic richness, DNA sequence ratio, and colonisation level of G-AMF and M-FRE communities, using partial RDA (for compositional variation) and LMM (for other community attributes). The test statistic is pseudo-F (without DFs) for RDA and the likelihood-ratio statistic (χ^2^) for LMM, with DF = 1. *EV* – explained variation. The effect direction is shown with ▼ for a decrease. *n.s.* – not significant, *n. a.* – not appropriate. Significance estimate with 0.05 < *p* < 0.10 is shown in parenthesis and interpreted as a non-significant outcome of the test

	*N* addition		*P* addition		*N*: *P* interaction	
	Test EV [%]	Effect description	Test EV [%]	Effect description	Test EV [%]	Effect description
Variation of G-AMF community	F = 3.9, *p* < 0.001 0.7%	Figure 1a	F = 1.7, *p* = 0.006 0.2%	Figure 2a	F = 0.8, n. s. < 0.1%	n. a.
Variation of M-FRE community	F = 4.4, *p* = 0.002 0.9%	Figure 3a	F = 1.1, n. s. < 0.1%	n. a.	F = 1.5, n. s. 0.1%	n. a.
α-diversity of G-AMF community	χ^2^ = 5.1, *p* = 0.024 1.5%	▼ 18%	χ^2^ = 1.5, n. s. 0.4%	n. a.	χ^2^ < 0.1, n. s. < 0.1%	n. a.
Richness of G-AMF community	χ^2^ = 5.3, *p* = 0.022 1.6%	▼ 10%	χ^2^ = 1.8, n. s. 0.5%	n. a.	χ^2^ = 0.1, n. s. < 0.1%	n. a.
α-diversity of M-FRE community	χ^2^ = 2.2, n. s. 0.6%	n. a.	χ^2^ = 0.8, n. s. 0.2%	n. a.	χ^2^ = 1.4, n. s. 0.4%	n. a.
Richness of M-FRE community	χ^2^ = 7.4, *p* = 0.007 1.9%	▼10%	χ^2^ = 0.7, n. s. 0.1%	n. a.	χ^2^ = 0.5, n. s. 0.1%	n. a.
G-AMF/M-FRE ratio	χ^2^ = 10.4,*p* = 0.001 3.5%	▼42%	χ^2^ = 2.0, n. s. 0.6%	n. a.	χ^2^ = 2.2, n. s. 0.6%	n. a.
G-AMF root colonisation	χ^2^ = 0.2, n. s. 0.8%		χ^2^ = 0.2, n. s. 0.1%	n. a.	χ^2^ = 0.1, n. s. < 0.1%	n. a.
M-FRE root colonisation	χ^2^ = 0.2, n. s. < 0.1%		χ^2^ = 2.9, n. s. (*p* = 0.088) 1.1%	n. a.	χ^2^ = 0.6, n. s. 0.2%	n. a.

**Fig. 5:**
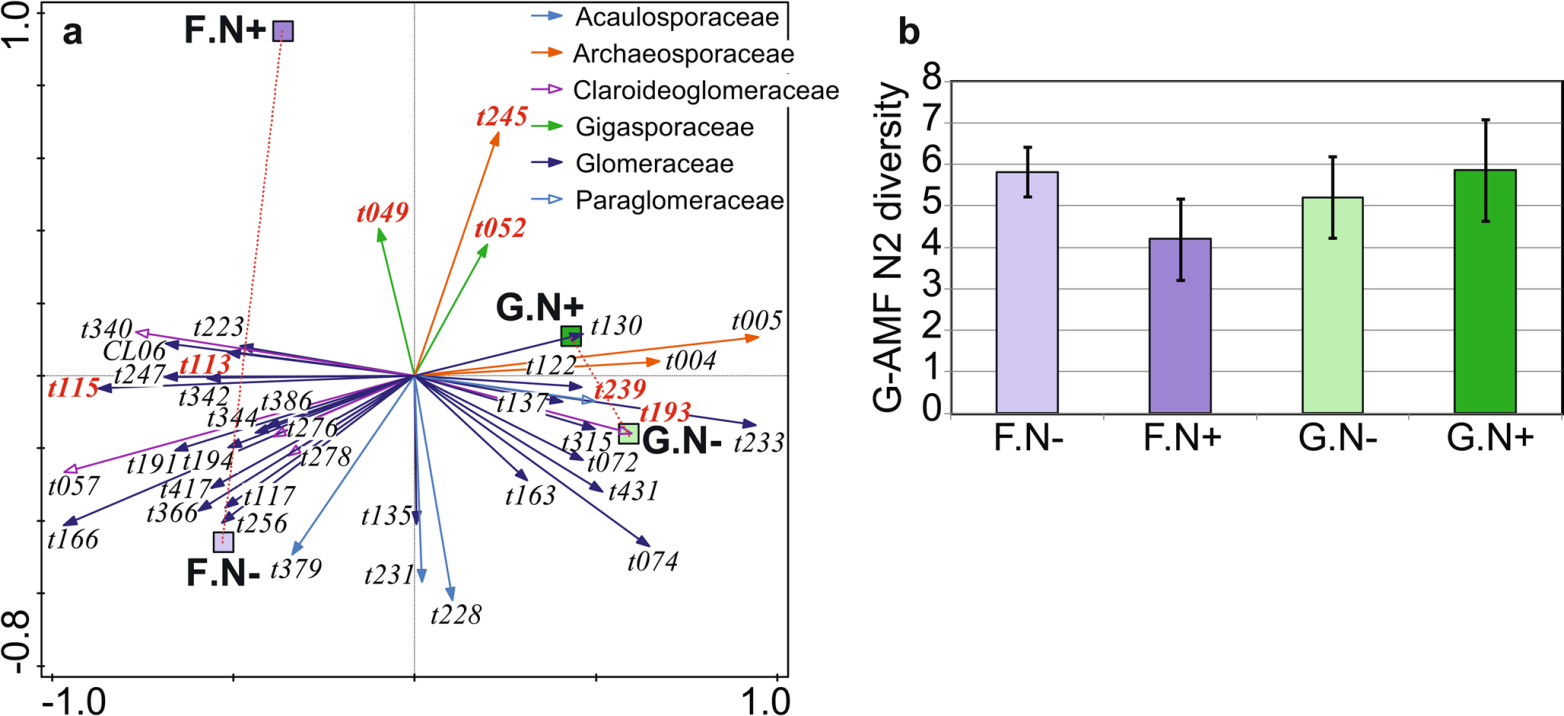
The effects of N addition (N- vs. N+), host species functional group (grass G, forb F), and their interaction on G-AMF community composition (**a**) and N2 diversity (**b**). The biplot of partial RDA (**a**) shows only the G-AMF VTXs strongly related to explanatory variables (based on t-value biplots), representing 60.1% of analysed DNA sequences. The arrow attributes show the assignment of VTXs to G-AMF families (the key is included within subplot **a**). VTX red bold labels indicate morphotaxa known from cultivation. Dotted red lines represent the compositional changes of G-AMF community with N addition in the roots of forb or grass species. Subplot **b** shows the average N2 diversity (with 95% confidence intervals) of G-AMF community for each combination of functional group and N addition. N- and N+ groups differ in N2 diversity only for the forbs (*p* = 0.025). For additional details about interpreting the ordination biplot (**a**) see Appendix EA4

When examining the variation in the fungal community response to N addition across host species, we found that the change in G-AMF community composition due to N addition was more substantial in forbs (Fig. 5a) and that G-AMF richness and α-diversity only decreased with N addition in forbs (Fig. 5b). There were no significant differences among host species in the response of the G-AMF community to P addition, and the response of the M-FRE community to N or P addition did not differ among host species, except for small differences in OTU richness due to N addition. Appendix EA7 provides additional information.

### Effects of background nutrient availability on fungal symbionts

The G-AMF community composition varied significantly, but to a small extent, compared to N and P background availability (Table 3). The relative abundance of 27 VTXs changed strongly with N availability based on a t-value biplot. Among the dominant G-AMF taxa (with > 1% of DNA sequences), two members of the Glomeraceae family (t074 and t219) and one from the Archaeosporaceae family (t005) had a higher relative abundance at sites with a low background N availability, while the relative abundances of t057 (Claroideoglomeraceae family), t113 (related to *Rhizophagus irregularis*), and three other Glomeraceae taxa (t108, t163, and t166) were higher at sites with a higher N availability (Fig. 1b). Seventeen VTXs responded strongly to the variation in background P availability. Among the dominant G-AMF taxa, only t074 (Glomeraceae) had a higher relative abundance at sites with a low P availability, and the relative abundance of five taxa, all from rhizophilic guild families (Claroideoglomeraceae – t057, t225; Glomeraceae – t212, t315; Paraglomeraceae – t281), was higher at sites with a higher P availability (Fig. 2b).

M-FRE community composition did not vary with background N availability (Table 3). However, there was higher M-FRE richness at sites with a higher background P availability (Fig. 4c; Table 3); richness increased by 7%, on average, with a 10% increase in P availability. Moreover, M-FRE community composition changed significantly, albeit to a small extent, with varying P availability (Table 3). Four M-FRE OTUs responded strongly to increasing P availability, mostly by increasing the relative abundance, including Otu004, which was the most dominant (Fig. 3b).

**Fig. 6:**
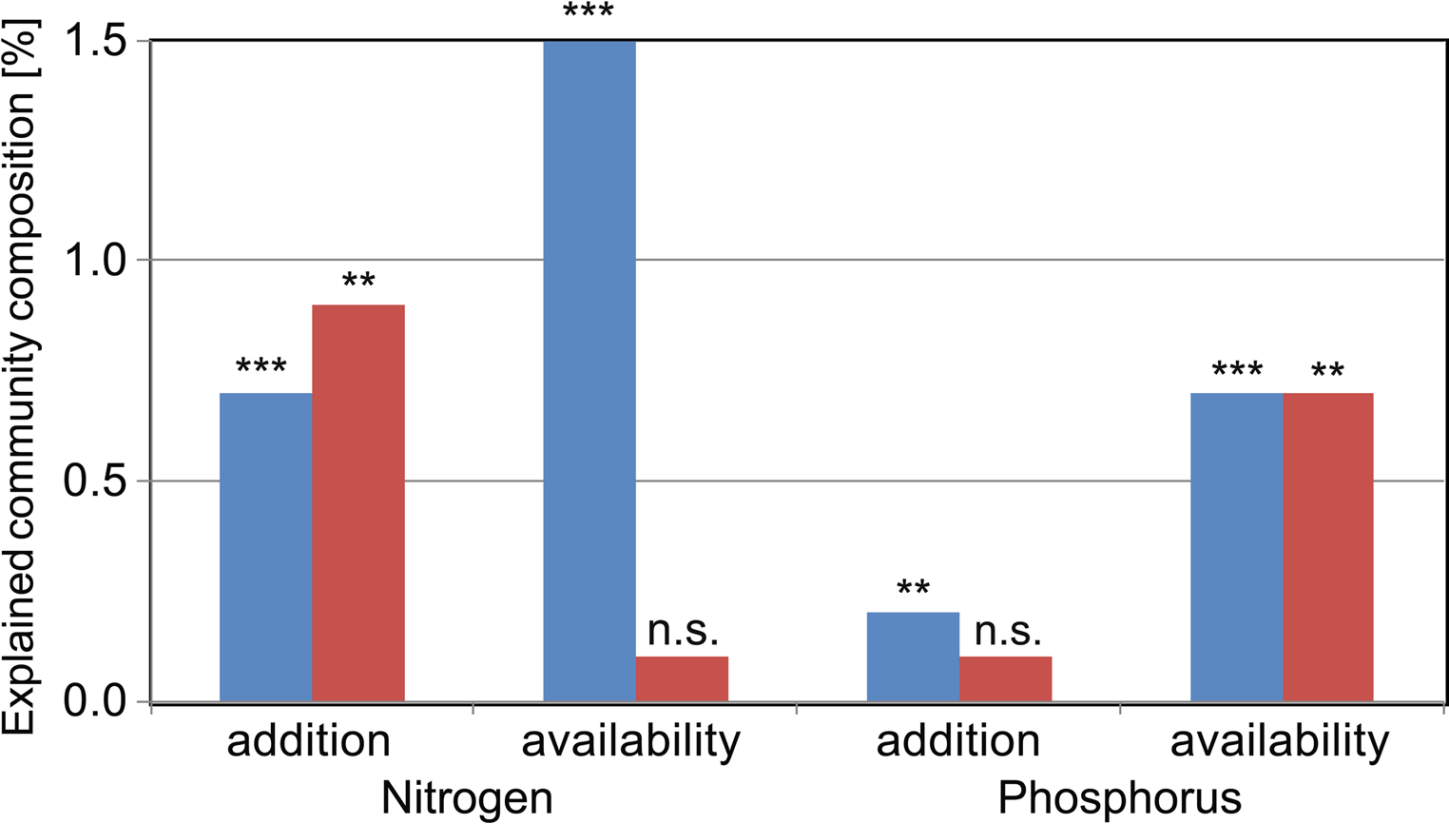
The impact of N and P background availability or addition on the G-AMF (blue bars) or M-FRE (red bars) community composition, quantified by adjusted coefficient of determination (R^2^_adj_). Each column corresponds to a RDA model (from Tables 2 and 3) and the text near the bar top summarises the effect significance (***– *p* < 0.001, ** – 0.01 > *p* > 0.001, n. s. – *p* > 0.05)

The effect of background nutrient availability was larger than that of nutrient addition for G-AMF community variation for both N and P. For M-FRE community, availability had larger effect only for P (Tables 2 and 3; Fig. 6), while in the case of N only its addition had a significant effect (Fig. 6). Also the background availability or addition of N had a larger impact than the corresponding predictors for P (Fig. 6). Overall, the variation explained in the community composition by nutrient availability or by nutrient addition was quite small.

**Table 3:** Summary of the statistical models examining the effects of N availability, P availability, and of the interaction between N addition and P availability on the composition, α-diversity (Hill’s N2 index), taxonomic richness, DNA sequence ratio, and colonisation level of G-AMF and M-FRE communities, using partial RDA (for compositional variation) and LMM (for other community attributes). The test statistic is the pseudo-F (used without DFs) for RDA and the likelihood ratio statistic (χ^2^) for LMM, with DF = 1. *EV* – explained variation. The effect direction is shown for univariate responses with ▼ for decrease and ▲ for increase. *n. s.* – not significant, *n. a.* – not appropriate; *IQR* – inter-quartile range. Significance estimates with 0.05 < *p* < 0.10 are shown in parentheses and interpreted as a non-significant outcome of the test

	*N* availability		*P* availability		*N* addition : *P* availability interaction	
	Test EV [%]	Effect description	Test EV [%]	Effect description	Test EV [%]	Effect description
Variation of G-AMF community	F = 6.3, *p* < 0.001 1.5%	Figure 1b	F = 3.5, *p* < 0.001 0.7%	Figure 2b	F = 2.3,*p* = 0.001 0.4%	Figure 7a
Variation of M-FRE community	F = 1.3, n. s. <0.1%	n. a.	F = 4.1, *p* = 0.006 0.7%	Figure 3b	F = 2.7,*p* = 0.014 0.5%	Figure 7b
α-diversity of G-AMF community	χ^2^ = 0.4, n. s. 0.2%	n. a.	χ^2^ = 2.2, n. s. 1.1%	n. a.	χ^2^ = 4.2,*p* = 0.041 1.2%	see ^A^
Richness of G-AMF community	χ^2^ = 1.1, n. s. 0.6%	n. a.	χ^2^ = 1.7, n. s. 0.8%	n. a.	χ^2^ = 1.8, n. s. 0.5%	n. a.
α-diversity of M-FRE community	χ^2^ = 0.1, n. s. < 0.1%	n. a.	χ^2^ = 0.1, n. s. < 0.1%	n. a.	χ^2^ = 1.2, n. s. 0.3%	n. a.
Richness of M-FRE community	χ^2^ = 0.2, n. s. < 0.1%	n. a.	χ^2^ = 7.4, *p* = 0.007 4.1%	▲ 7% with 10% increase	χ^2^ = 9.0,*p* = 0.003 2.2%	see ^B^
G-AMF/M-FRE ratio	χ^2^ = 3.8, *p* = 0.050 2.3%	see ^C^	χ^2^ = 2.5, n. s. 1.7%	n. a.	χ^2^ = 2.7, (*p* = 0.098) n. s. 0.8%	n. a.
G-AMF root colonisation	χ^2^ = 0.4, n. s. < 0.1%	n. a.	χ^2^ = 1.3, n. s. 0.9%	n. a.	χ^2^ = 2.3, n. s. 1.7%	n. a.
M-FRE root colonisation	χ^2^ = 0.3, n. s. < 0.1%	n. a.	χ^2^ = 0.2, n. s. < 0.1%	n. a.	χ^2^ = 1.0, n. s. 0.1%	n. a.

^A^ Without N addition, there was no significant change with background P availability, but G-AMF diversity ▲ by 17% with a 10% increase of P availability when N was added

^B^ Without N addition, M-FRE richness▲ by 3.0% with 10% increase of P availability, but with N added, richness ▲ by 12.6% with 10% increase of P availability

^C^ ▼ by 11.0% with a 10% increase of N availability

### Interaction between N addition and P availability

**Fig. 7:**
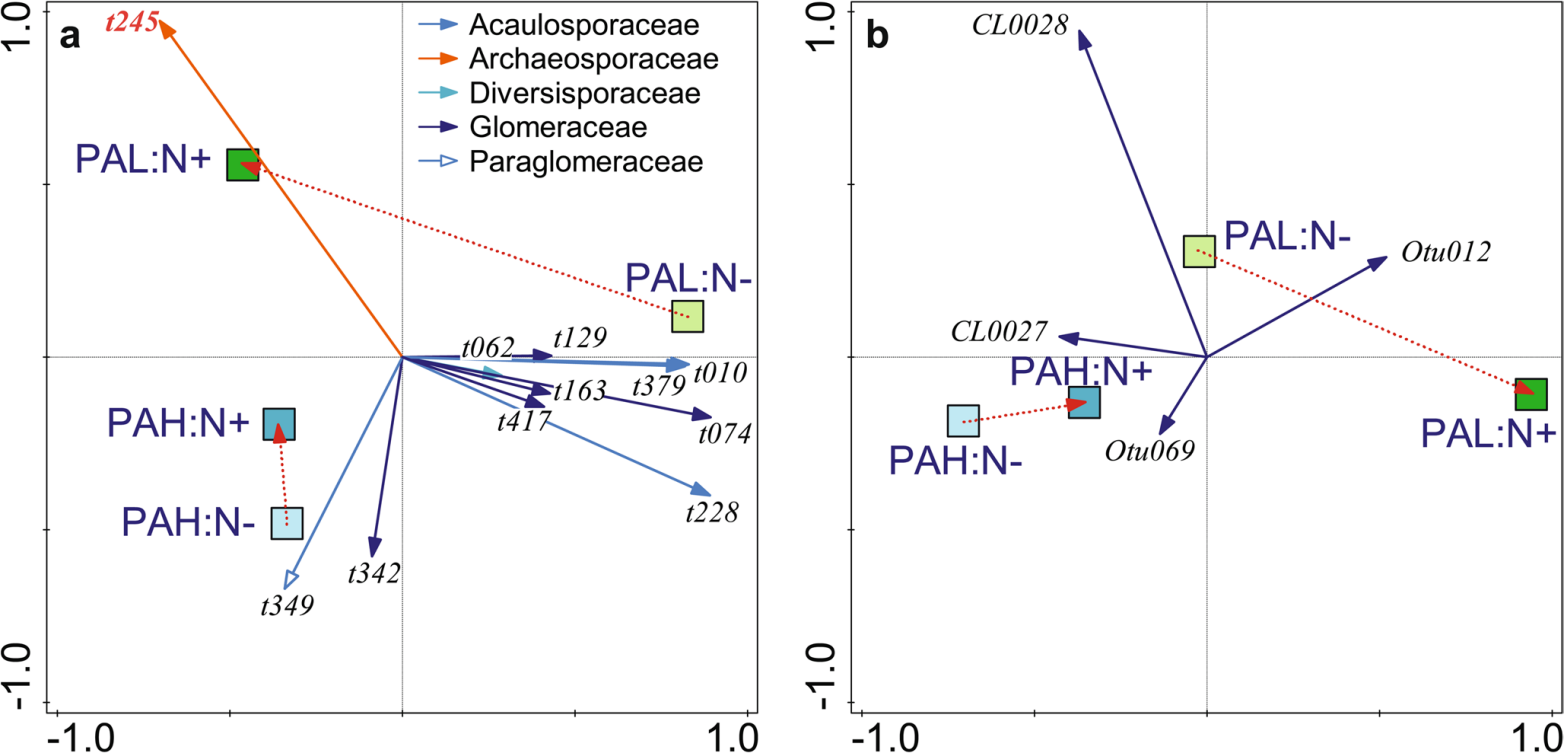
The effects of N addition, background P availability, and their interaction on G-AMF (**a**) and M-FRE (**b**) community composition, shown as biplots of partial RDA. Only the G-AMF/M-FRE taxa strongly related to the interaction term (based on a t-value biplot) are shown, representing 29.4% (**a**) and 20.0% (**b**) of analysed DNA sequences in respective datasets. In **a**, the arrow attributes show the assignment of VTXs to G-AMF families (the key is in the upper right corner). The Archaeosporaceae VTX t245 with a red bold label is related to a morphotaxon known from the cultivation as *Archaeospora trappei*. For clarity, the interaction is visualised using four square centroids representing groups of sites combining low (PAL) or high (PAH) P availability with N addition (N+) or no N addition (N-). Quantitative P availability was categorised into two groups (PAL vs. PAH) using median (0.73) as a threshold. Dotted red arrows represent the compositional change of PAL or PAH plots after N addition, with the arrow length estimating the size of compositional change. For additional details about interpreting ordination biplots, see Appendix EA4

The composition of the G-AMF and M-FRE communities responded differently to N addition depending on the background P availability (Table 3). Figure 7 summarises the community changes. The impact of N addition on community composition (two red, dotted arrows in each subplot) was larger at sites with low P availability, both for G-AMF and M-FRE.

When we explored the effects of N addition on G-AMF community separately for sites with a lower vs. higher P background availability (delimited by the P availability median value), we found that N addition had a significant effect on the community composition (R^2^_adj_ = 2.4%, pseudo-F = 5.8, *p* < 0.001) at the sites with low P availability but not at sites with higher P availability (R^2^_adj_ = 0.1%, pseudo-F = 1.2, n.s.). We found 16 G-AMF VTXs with a strong response to N addition at sites with a low P availability. Among these, five VTXs from the Acaulosporaceae family (t010, t013, t228, t231, and t379) declined with N addition, but they all had relatively low abundances. Among the four VTXs with a higher relative abundance (> 1% of all DNA sequences), only one (t245 from Archaeosporaceae) was more abundant in fertilised plots, and the remaining three, all belonging to Glomeraceae, had higher abundances in plots not fertilised with N.

The dependency of the response to N addition on the background P availability was also significant for the M-FRE community (Fig. 7b; Table 3). N addition had no effect at sites with a higher P availability (R^2^_adj_ = 0.4%, pseudo-F = 1.7, n.s.), whereas it was important for sites with a lower P availability (R^2^_adj_ = 1.7%, pseudo-F = 4.5, *p* < 0.001).

The interaction between N addition and background P availability also affected the α-diversity of the G-AMF community (Table 3). In the absence of N addition, there were no significant changes in α-diversity with P availability, while when N was added, plots at sites with a higher P availability had a greater α-diversity than the plots at sites with a low P availability. For the M-FRE community, N addition increased the positive change in OTU richness with background P availability (Table 3). We found no significant interaction pattern for G-AMF richness or M-FRE α-diversity.

## Discussion

### G-AMF associated with forbs are more responsive to N addition

In both groups of fungal symbionts, we found important differences among the host plant species in the community composition and diversity as well as in root colonisation. The differences in the G-AMF and M-FRE community composition between (C3) grasses and forbs dominated in the host species comparisons. Systematic differences between forbs and grasses in the response to N addition were found only for G-AMF composition, diversity and richness (Fig. 5). The observed response of G-AMF diversity and richness (no change in grasses, decline in forbs) is consistent with the results of Zheng et al. (2018). Forb hosts invest more resources to their symbiotic fungi than grasses (Unger et al. 2016) and harbour a more phylogenetically diverse spectrum of G-AMF (Davison et al. 2020), but our results suggest that they also reduce their C investment more readily than grasses with increased N availability. In summary, our results confirm hypothesis H1 but only for G-AMF.

### Fertilisation with N has a stronger effect than P fertilisation

The G-AMF community composition changed more with N addition than with P addition, and the M-FRE community composition only changed significantly with N addition (Fig. 6). However, even for the larger N effects, the extent of the compositional change was small (Table 2; Fig. 6). G-AMF diversity (and richness) decreased with N addition but only in the roots of forbs. The different responses of the two functional groups, observed here under field conditions, agree with our previous findings from a greenhouse experiment (Šmilauer et al. 2021b), but the results of that study were based on combined N and P fertilisation. Our findings also partly agree with the results of Chen et al. (2014), who applied N and P factorially and found that N addition modified community composition after 6 years of treatment, in contrast to P, which changed colonisation levels. Overall, our results support hypothesis H2.

We also observed a lower G-AMF/M-FRE ratio after N addition, due to an increased abundance of M-FRE DNA sequences, but there was no corresponding response to N or P addition in root colonisation for either fungal group, in contrast to other published studies in which nutrient addition reduced the total colonisation (Camenzind et al. 2014; Han et al. 2020; Šmilauer et al. 2025). However, our results are consistent with those of Jumpponen et al. (2005), who also reported that N addition did not affect G-AMF root colonisation but changed the G-AMF community composition. In a study from the Australian woodlands, Albornoz et al. (2024) found that M-FRE were more resistant to high pollution with N (and P) compared to G-AMF, which agrees with our results showing a decrease in the G-AMF/M-FRE ratio with N addition.

### The effects of background nutrient availability are stronger than those of fertilisation

G-AMF community composition varied significantly with the background nutrient availability estimated for individual sites, and this response was stronger than the community response to the short-term addition of nutrients (Fig. 6). This is likely a consequence of longer-term co-adaptation of the plant and G-AMF communities to available nutrient resources (Klironomos 2003; Johnson et al. 2010), which affects the pool of fungal symbionts colonising seedlings (Antunes et al. 2012). M-FRE community composition only responded to the variation in background P availability, although it was only affected by N addition (Fig. 6). Similarly, M-FRE taxonomic richness declined either with N addition or with a lower P availability. We also demonstrated the interaction of the effects of these two factors (P availability and N fertilisation) on community composition and diversity (G-AMF) or richness (M-FRE), as discussed below. Overall, background nutrient availability was a more decisive factor for fungal community composition than nutrient addition, supporting hypothesis H3.

The estimated N and P availabilities and the observed effects of N and/or P addition on the increase in aboveground biomass (Table EA5 in Appendix EA6) suggest that our experimental grasslands were co-limited by N and P, which seems to be a common feature among grasslands throughout the world (Fay et al. 2015). However, the fungal symbionts mostly responded to N addition, with no detected response to P addition except a small change in G-AMF community composition (Fig. 2a; Table 2).

The negative effects of P addition on the G-AMF community were strongest for the relative abundance of Glomeraceae VTX t074, which was the most abundant taxon in the G-AMF dataset (19.3% of all DNA sequences, Table EA2 in Appendix EA5). N addition decreased its relative abundance (Fig. 1a), and it was also less abundant at sites with higher background N and P availabilities (Figs. 1b and 2b). In a previous study, we found that VTX t074 was more abundant in grass hosts than in forbs (Figs. 1a and 2a in Šmilauer et al. 2025), and its abundance substantially declined in soil transferred to controlled greenhouse conditions (Fig. 6 in Šmilauer et al. 2025). Therefore, we suggest that VTX t074 is one of the G-AMF workhorse taxa for nutrient exchange, but it has not been identified as such in studies performed under controlled conditions.

The experimental N addition reduced the taxonomic richness of the G-AMF and M-FRE communities, but we observed no changes in their diversity or richness along the background N availability gradient. Higher N availability may select fungal symbionts (over a longer temporal scale) that provide greater host protection against soil pathogens (Maherali and Klironomos 2007), as a higher nutrient availability also increases the abundance of pathogenic fungi in the soil (Lekberg et al. 2021).

Root colonisation by either G-AMF or M-FRE did not change significantly with N or P addition or with the background availability of those nutrients, but the independently estimated G-AMF/M-FRE ratio strongly decreased with N addition, primarily due to larger M-FRE DNA sequence counts, and the ratio was also slightly lower (*p* = 0.050, Table 3) at sites with a higher background N availability. Facultatively saprotrophic M-FRE (Field et al. 2019) might be able to colonise roots even with a reduced C supply from the host. On contrary, G-AMF are entirely dependent on C supply from the host and are therefore expected to limit their hyphal growth in roots as the host reduces C provision under a surplus of traded nutrients (Johnson 2010; Treseder et al. 2018). Such explanation was supported by observed responses of M-FRE and G-AMF colonisation to N addition: both groups responded by a non-significant decrease with a larger extent in G-AMF (-12%) than in M-FRE (-6%). Grman and Robinson (2013) demonstrated the limitation of G-AMF abundance by N availability using hyphal length as a measure of colonisation. Our study could not confirm the change in root colonisation, but we used young seedlings that depend more on the presence of fungal symbionts (Van der Heijden 2004) and therefore might not respond strongly to nutrient cues.

Some VTXs of the Glomeraceae family showed contrasting responses to nutrient background availability and addition (Fig. 1a and b, and 2b). This agrees with previous reports of functional diversity among Glomeraceae taxa (Klironomos 2000; Šmilauer et al. 2025). We observed similar inconsistencies in the Acaulosporaceae family, particularly in its response to P availability gradients (Fig. 2b), and this matches the reported varying preferences of Acaulosporaceae taxa for P availability in woodland sites of Western Australia (Albornoz et al. 2024). Similar to families, we also found no consistency in the response of G-AMF guilds (as defined by Weber et al. 2019) to nutrients. Whether we looked at the responses of G-AMF VTXs to N or P addition or to background N or P availability (Figs. 1 and 2) or even the VTXs defining the interaction between background P availability and N addition (Fig. 7), a particular response included VTXs assigned to two or even all three G-AMF guilds. These inconsistencies show that gross differences among hyphal colonisation strategies, on which the guild definition is based, do not provide an appropriate starting point for understanding niche differentiation among G-AMF (Camenzind et al. 2024).

### The effects of N addition vary depending on the background P availability

We demonstrated an interactive effect between N addition and background P availability on the composition of G-AMF and M-FRE communities (Table 3), as predicted by hypothesis H4. We found that the fungal community composition did not change much after N addition at sites with a high background P availability. The lack of an impact of N with sufficient available P is apparent in Fig. 7 based on the different lengths of the arrows connecting the centroids of community samples from plots with or without added N and for sites with low and high P availability.

Although the G-AMF taxonomic diversity and M-FRE taxonomic richness declined with N addition (Table 2), they were higher at sites with a high P availability only with N addition. This is contradictory to previously published results (Johnson 2010) in which G-AMF diversity declined with N addition at P-rich sites compared to P-limited ones. When we examined the identity of taxa positively responding to the P availability gradient, the increase in G-AMF diversity (Fig. 2b) was mainly due to the taxa of the rhizophilous guild (from Glomeraceae and Claroideoglomeraceae families), which are hypothesised to provide better protection to plant roots against fungal pathogens than fungi from other guilds (Maherali and Klironomos 2007; Weber et al. 2019). Notably, our study demonstrates a pattern of dependence for the fungal community response to N fertilisation based on the P availability already known in G-AMF (Johnson 2010; Grman and Robinson 2013; Han et al. 2020) as a new finding for M-FRE symbionts. For M-FRE, the interaction between N addition and P availability was significant for OTU richness but not N2 diversity, which is likely an artefact of very low evenness in the M-FRE dataset. Two OTUs (OTU004 and OTU012, former one already known as a mycorrhizal symbiont – Bidartondo et al. 2011) represent over 75% of all DNA sequences (Table EA3 in Appendix EA5), thus reducing the variability of N2 estimates across samples.

### Advantages and limitations of the chosen experimental approach

This study focused on a stage of mycorrhizal symbiosis when it becomes established in host roots and the vacant living space in the seedling roots likely enabled a broader spectrum of initial fungal colonisers (Bever 2015). We examined how this stage is affected by N and P addition as well as by the pool of available fungal symbionts already filtered by the background availability of N and P.

Except for seedling germination under laboratory conditions, all steps were performed in the field, avoiding cultivation-based procedures, such as the transfer of field-based fungal communities into a cultivation experiment, as done e.g. by Antunes et al. (2012). In this way, our approach prevented the substantial filtering of symbiotic fungi by cultivation conditions (Šmilauer et al. 2021b). Therefore, we report the fungal community response to fertilisation under realistic circumstances, with local fungal symbionts adapted to local environmental conditions.

When experimenting with nutrient addition, we could not distinguish the direct effects of increased nutrient availability and their effects mediated through an increase in the aboveground biomass under field conditions. In nutrient-rich plots, the light conditions were modified (Appendix EA6), which likely affected the amount of C provided by seedlings to fungal symbionts and the fungal community attributes due to presumably increased competition (Grman and Robinson 2013; Knegt et al. 2016).

## Conclusions

- G-AMF communities in forb roots responded to N addition by greater changes in their composition compared to grasses, but there were no differences in the response of their fungal symbionts to P addition between host species.
- The effects of N addition on G-AMF and M-FRE community composition and richness and the effects of background N availability on G-AMF community composition were stronger than the corresponding P-related effects.
- The background P availability and N availability (for G-AMF community) represented more effective predictors of the G-AMF and M-FRE community composition and M-FRE richness than P or N addition.
- The effects of inorganic N addition on fungal symbionts depended on the background P availability at the site, and we demonstrated this relationship not only for G-AMF but also for M-FRE symbionts.
- Overall the effects of long-term nutrient availability and experimental nutrient addition explained small amount of variation in community composition and diversity for both fungal groups, compared with among-site differences or host species effect.

## Supplementary Information

Below is the link to the electronic supplementary material.

Supplementary Material 1

## Data Availability

Data from Illumina sequencing and all the additional datasets used in this manuscript are accessible on *zenodo.org* platform (Košnar et al. 2025b). DNA sequences are available on GenBank under the accessions PX244395 – PX244520.
