# Supplementary material for: Phosphorus availability modifies the responses of Glomeromycotina and Mucoromycotina to nitrogen addition
Source: Mycorrhiza. 2026 Mar 26;36(2):11. doi: 10.1007/s00572-026-01256-5 (PMC13021758; doi:10.1007/s00572-026-01256-5)
Supplement: Supplementary file 1 — Supplementary Material 1 [file 572_2026_1256_MOESM1_ESM.docx]

# Supplementary Information

Košnar J, Šmilauerová M, Pecková T, Šmilauer P (2026): Phosphorus availability modifies the responses of Glomeromycotina and Mucoromycotina to nitrogen addition. Mycorrhiza XXX

# List of electronic appendices

| EA1 | Experimental and sampling design | 2 |
| --- | --- | --- |
| EA2 | DNA analyses | 9 |
| EA3 | Bioinformatic analysis | 12 |
| EA4 | Models of statistical analysis | 20 |
| EA5 | Overview of G-AMF VTX and M-FRE OTU | 24 |
| EA6 | Soil properties, biomass of seedlings and plant community | 33 |
| EA7 | Interaction between nutrient addition and host plant identity | 43 |

# Appendix EA1 – Experimental and sampling design

Table EA1 provides geographical locations, altitude and basic vegetation characteristics of all 12 sites. The sites were located in the southern part of the Czech Republic, with mean annual temperature 10.4°C and annual sum of precipitation 637 mm. The bedrock is mostly metamorphic rock granulite and soils are acidic Cambisols.

The sites were selected based on a wider screening of 25 locations representing actively farmed grasslands with dominant perennial grasses and forbs, excluding highly productive resown grasslands with a single dominant grass species. The sites were selected to span the observed gradients of N and P availability, estimated from soil analyses. The eight most dominant plant species (summing up to 50% of total aboveground cover across all species and plots) in selected 12 sites were (in order of descending relative abundance) *Plantago lanceolata*, *Galium boreale*, *Festuca rubra*, *Trifolium pratense*, *Alopecurus pratensis*, *Anthoxanthum odoratum*, *Trifolium medium*, *Achillea millefolium* (nomenclature based on Kaplan et al. 2019). Vegetation composition of individual experimental plots is available in the source data for this study (Košnar et al. 2025).

In March 2022, we fenced at each site an area of 6 x 5 m (using wire mesh with the height of 1.5 m) to protect experimental plots from browsing and rooting by large animals. Within the fenced area, we established eight experimental plots 1 x 1 m arranged into two rows and four columns and separated by 0.5 m wide buffer zones. Four nutrient addition treatments (control, N, P, N+P) were randomly assigned to individual plots within each row. Consequently, each site represented one complete randomised block with two replicates of each fertilisation treatment.

**Table EA1** – Overview of experimental sites. *Coordinates* show the degrees of World Geodetic System (WGS 84), the *Aboveground biomass* approximates productivity of plant community and was collected in June 2022, the *Proportion of forbs* (i.e. dicotyledonous herbs) was subjectively estimated as the percentage of the total vegetation cover.

| **Site number** | **Coordinates** | | **Altitude** | **Aboveground biomass** | **Proportion  of forbs** |
| --- | --- | --- | --- | --- | --- |
|  | N [degrees] | E [degrees] | [m a.s.l.] | [DW g . m^-2^] | [% of cover] |
| **1** | 48.9279075 | 14.3345544 | 572 | 371.2 | 82 |
| **2** | 48.9282447 | 14.3351650 | 563 | 304.0 | 78 |
| **3** | 48.9311392 | 14.3380950 | 515 | 167.1 | 60 |
| **4** | 48.9320097 | 14.3399511 | 498 | 484.2 | 78 |
| **5** | 48.9285039 | 14.3415900 | 523 | 295.5 | 90 |
| **6** | 48.9317550 | 14.3355228 | 534 | 239.7 | 38 |
| **7** | 48.9284150 | 14.3421800 | 521 | 418.6 | 53 |
| **8** | 48.9998419 | 14.5831711 | 542 | 363.1 | 73 |
| **9** | 48.9994194 | 14.5834233 | 541 | 200.5 | 75 |
| **10** | 48.9887864 | 14.6082094 | 506 | 357.2 | 83 |
| **11** | 48.9889694 | 14.6079681 | 501 | 435.0 | 96 |
| **12** | 48.9899867 | 14.6079533 | 506 | 247.4 | 75 |

Fig. EA1 shows the scheme of treatment and sampling arrangement in a single experimental plot. Each plot was fertilised across its whole area with 2 L of tap water, containing dissolved 7.6 g K_2_HPO_4_ for P and N+P plots, and 20 g NH_4_NO_3_ for N and N+P plots; with no addition for control plots. Fertilisation was repeated three times before planting bait seedlings –April and September 2022 and March 2023.


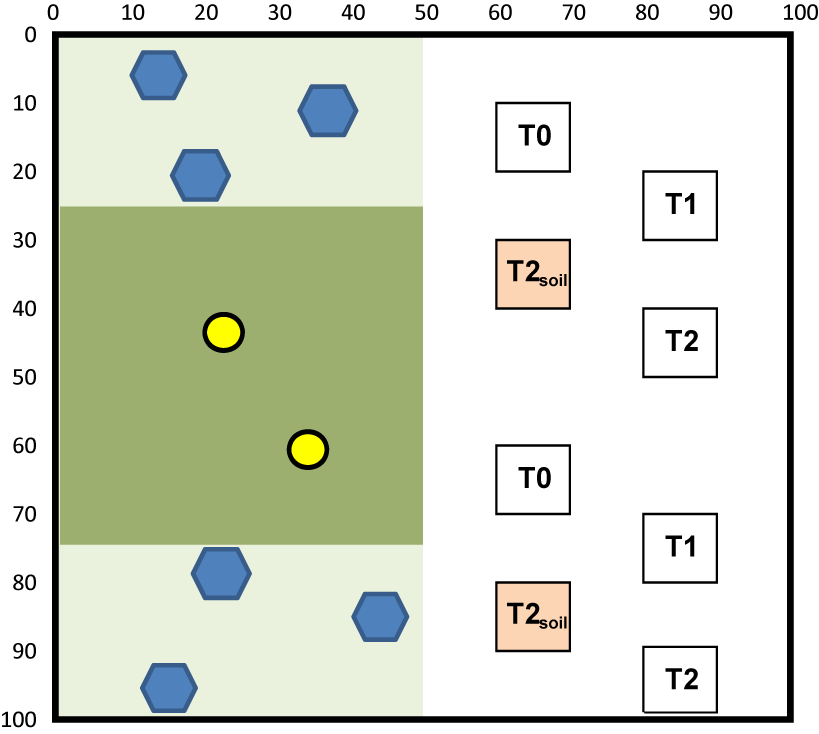


**Fig. EA1** – Schematic overview of treatment and sampling of individual plot. Numbers at the top and left edges represent coordinates in cm. Dark green area was used for sampling plant biomass aboveground and belowground (using two soil cores at yellow circle positions). Blue hexagons represent six groups of planted seedlings. Sampling locations in the right half of the plot belong to another experiment performed at the same sites and were not used in this study except the T2_soil_ locations of soil collection for chemistry in 2024.

We planted experimental seedlings into each of 96 plots (at 12 sites), with 12 replicates per plot for each species (i.e., 12 * 8 * 12 = 1152 seedlings per species) in early April 2023. Four grassland plant species were selected (plant nomenclature follows Kaplan et al. 2019): *Plantago lanceolata* (PL) and *Centaurea jacea* (CJ) represented forb species, *Anthoxanthum odoratum* (AO) and *Poa angustifolia* (PA) represented grass species. Additionally, we also planted seedlings of a forb *Betonica officinalis* (BO) to all plots at sites 2, 5, 10 and 12 (i.e., 4 * 8 * 12 = 384 seedlings for this species). All chosen species were naturally occurring across multiple experimental sites (occurring in 6 – 12 sites, BO in 3 sites) and we used them in previous studies (e.g. Šmilauer et al. 2021). We used 1 – 2 week old seedlings germinated on a sterile sand from surface-sterilised seeds that originated from the area of sites 10 – 11. Seedlings were planted into six groups within each plot (each group with two replicates of planted species), forming in each group a ring around a central colour plastic stick, which enabled us to quickly locate the seedlings in the vegetation. Each seedling was planted within a small rubber ring (inner diameter 1 cm), fixed to soil surface with a split pin stuck into the ground.

In addition to whole plot fertilisation, we also fertilised the seedlings with 80 mL of water per seedling group, with dissolved 0.52 g of K_2_HPO_4_ in P and N+P plots, and 1.35 g of NH_4_NO_3_ in N and N+P plots. Seedlings were fertilised 10 days after planting and then after another four weeks. We collected surviving seedlings 11 weeks after planting (in late June 2023) by excavating soil blocks, each one containing a single group of seedlings; individual plants were removed from the blocks in laboratory. Seedling roots were carefully washed and the roots belonging to the same plant species from each plot were pooled (0 – 12 seedlings, average 6.3). This resulted in 356 root samples for molecular analyses (remaining 60 species–plot combinations were not collected due to seedling mortality). Pooled roots were dried six hours at 60°C and those used for molecular analysis frozen before further processing. We also pooled the aboveground parts, combining individuals of the same species from both plots with particular treatment and then usually also from two or three nearby sites, to arrive at acceptable dry weight value for chemical analyses of nutrient contents.

Seedling roots were primarily used to extract DNA to identify present G‑AMF VTX and M‑FRE OTU and to estimate their relative abundance. However, when sufficient amount of roots was available, we used part of them also for light microscopy to estimate the colonisation rates of G‑AMF and M‑FRE (200 samples). Number of analysed samples varied between fertilisation treatments (Ctrl – 77, N – 25, P – 81, N+P – 17) and also between host species (AO – 65, BO – 27, CJ – 35, PA – 27, PL - 46). For the microscopy, roots were stained with Chlorazol Black E using the protocol of Vierheilig et al. (2005). The colonisation extent was estimated using Olympus BX-50 microscope (Olympus, Japan) at 200× magnification. For each root sample (pooled seedling roots of the same species and from the same plot), we visually estimated the percentage of root length colonised by G‑AMF and M‑FRE in 20 view fields and we also estimated the staining quality (on a scale from 0.0 to 1.0), which was then used as a relative weight for individual samples in statistical models. Data from individual view fields were aggregated at the sample level by arithmetic average. M‑FRE fungi were recognised based on their fine hyphae (<2 μm diameter) with small intercalary and terminal swellings and occasionally present “fan‑like” branching (Sinanaj et al. 2021).

We recorded plant community composition in each of 96 plots in early June 2023. Fertilisation of 1 × 1 m plots continued – after the collection of seedlings – in September 2023 and March 2024 as a part of another experiment. We then collected the soil for physico-chemical analyses in April 2024 (pooling soil cores taken from the depth 0 – 10 cm in two locations labelled T2_soil_ in Fig. EA1). We estimated soil acidity (pH, from a water slurry), available N in inorganic ions (N‑NH_4_ and N-NO_3_) extracted with KCl and analysed by flow injection analysis, and available P in orthophosphate form (P-PO_4_) using the method of Mehlich (1984). We further calculated the ratio of inorganic N to inorganic P.

We collected aboveground plant biomass and also the belowground biomass from the top soil layer (0 – 10 cm soil, using two pooled soil cores per plot, see Fig. EA1) in late May 2024 and analysed the biomass content of C, N and P. Data on N and P concentration in aboveground biomass were used in this study to estimate the background N availability and P availability, based on the proposal of Ostertag & DiManno (2016), using the formula *X_avail_ = [X_ctrl_] / [X_X+_]* , where *X* represents a macronutrient element (either N or P), *[X_X+_]* is the X concentration in the aboveground biomass of plots where *X* was added by fertilisation (this includes for both elements the plots where N and P were applied together), and *[X_ctrl_]* is the X concentration in the aboveground biomass of control plots with no added nutrients. These estimates of nutrient availability were used in analyses on log-transformed scale, so they effectively represent log-ratio changes of N or P concentration due to fertilisation. Note that our N and P availability estimates have opposite sign than the response ratios of Ostertag & DiManno (2016) and therefore represent the relative proportions of nutrient concentration under non-manipulated conditions in a comparison with the conditions where nutrient addition was applied for sufficiently long time (three seasons).

Additionally, we estimated the attenuation of photosynthetically active radiation (PAR) by aboveground vegetation cover, measured at the soil surface to represent the light available for the seedlings. The measurements were performed on a clear, radiant day in June 2023, using canopy analysis system SunScan SS1 (Delta-T Devices Ltd., Winster, UK), with measuring rod crossing the whole plot in its centre. Measured PAR intensity at the soil surface was related to PAR at the (non-shaded) upper surface of the aboveground biomass.

## References

Kaplan Y, Danihelka J, Chrtek J, Kirschner J, Kubát K, Štech M, Štěpánek J (2019) Key to the flora of the Czech Republic. (in Czech), Edition 2, 1168 pp., Academia, Prague

Košnar J, Šmilauerová M, Pecková T, Šmilauer P (2025) Dataset for paper “Background phosphorus availability modifies the response of Glomeromycotina and Mucoromycotina symbionts of plant seedlings to nitrogen fertilisation under field conditions. Public repository zenodo.org. https://doi.org/10.5281/zenodo.17617977

Mehlich A (1984) Mehlich 3 soil test extractant: a modification of Mehlich 2 extractant. Communications in Soil Science and Plant Analysis 15: 1409–1416. https://doi.org/10.1080/00103628409367568

Ostertag S, DiManno NM (2016) Detecting terrestrial nutrient limitation: a global meta-analysis of foliar nutrient concentrations after fertilization. Front Earth Sci 4: 23. https://doi.org/10.3389/feart.2016.00023

Sinanaj B, Hoysted GA, Pressel S, Bidartondo MI, Field KJ (2021): Critical research challenges facing Mucoromycotina ‘fine root endophytes’. New Phytol 232: 1528–1534. https://doi.org/10.1111/nph.17684

Vierheilig H, Schweiger P, Brundrett MC (2005) An overview of methods for the detection and observation of arbuscular mycorrhizal fungi in roots. Physiol Plantarum 125: 393–404. https://doi.org/10.1111/j.1399-3054.2005.00564.x

# Appendix EA2 – DNA analyses

## G-AMF detection

A 550-bp fragment of SSU rDNA was amplified by semi-nested PCR as described by Dumbrell et al. (2011), but the AML2 primer (Lee et al. 2008) was used as the AMF-specific primer. The first PCR amplification with the NS31 forward primer (Simon et al. 1992) and the AML2 reverse primer was run in a reaction mixture containing 0.7 μL of template DNA, 0.2 mM of each dNTP, 0.4 μM of each primer (Integrated DNA Technologies, Coralville, Iowa, USA), 0.1 U Phusion High-Fidelity DNA Polymerase (New England Biolabs, Ipswich, Massachusetts, USA), 1× Phusion HF buffer, and sterile water to make up a final volume of 5 μL. Each sample was amplified in three independent replicates with a final reaction volume of 15 μL. Amplifications were performed with an initial denaturation for 30 s at 98°C; followed by 35 cycles of 10s at 98°C, 30s at 68°C, and 15s at 72°C; and a final extension of 5 min at 72°C.

The second PCR was performed as described above, except that 0.5 μL of the first PCR product was used as a template, and the WANDA primer (Dumbrell et al. 2011) fused with sample-specific barcode sequence was used as a forward primer, and that the final volume of the reaction was increased to 10 μL. The barcode sequences were adopted from the Roche Extended Set of multiplex identifiers (MID; 454 Sequencing Technical Bulletin No. 005-2009; Roche, Basel, Switzerland). Cycling conditions were the same as for the first PCR, except that the annealing temperature was set to 60°C and only 10 cycles were used. Each sample was amplified in three independent replicates with a final reaction volume of 30 μL. The resulting products of the second PCR were pooled and purified using NucleoSpin Gel and PCR Clean-up kit (Macherey Nagel, Düren, Germany). The individual MID-barcoded samples were pooled and further purified using Agencourt AMPure XP beads (Beckman Coulter Brea, California, USA). We amplified successfully 327 out of 356 root samples entering the analysis. The resulting PCR amplicon pool was subjected to Illumina paired-end 2 × 300 bp sequencing performed at the Institute of Applied Biotechnologies (Prague, Czech Republic).

## M-FRE detection

A 280-bp fragment of SSU rDNA was amplified using a single-step PCR with the primer pair AMV4.5NF and AMDGR (Sato et al. 2005). The PCR was performed in a reaction mixture containing 1 μL of template DNA, 0.2 mM of each dNTP, 0.3 μM of each primer (Integrated DNA Technologies, Coralville, Iowa, USA), 0.5 U Taq Polymerase (Top-Bio, Prague, Czech Republic) in the manufacturer´s reaction buffer, and sterile water to make up a final volume of 10 μL. Amplifications were performed with an initial denaturation of 3 min at 95°C, followed by 40 cycles of 30 s at 95°C, 30 s at 55°C, and 30 s at 72°C, and then a final extension of 10 min at 72°C. Both forward and reverse rimers were fused with a sample-specific barcode sequence to allow sample identification in sequencing results. A set of 12 forward and 16 reverse barcoded primers, providing up to 192 possible unique dual combinations, was used. The barcode sequences were adopted from Roche’s Extended Set of MIDs (454 Sequencing Technical Bulletin No. 005-2009; Roche, Basel, Switzerland).

Each sample was amplified in three independent technical PCR replicates with a final reaction volume of 30 μL. The resulting PCR products were pooled and purified using the NucleoSpin Gel and PCR Clean-up kit (Macherey Nagel, Düren, Germany). We amplified successfully all 356 root samples entering the analysis. Individual barcode-tagged samples were pooled in equimolar concentrations and the pool was further purified using Agencourt AMPure XP beads (Beckman Coulter Brea, California, USA). The paired-end 2 × 250 bp Illumina sequencing was performed at the SEQme company (Dobříš, Czech Republic).

## References

Dumbrell AJ, Ashton PD, Aziz N, Feng G, Nelson M, Dytham C, Fitter AH, Helgason T (2011) Distinct seasonal assemblages of arbuscular mycorrhizal fungi revealed by massive parallel pyrosequencing. New Phytol 190: 794–804. https://doi.org/10.1111/j.1469-8137.2010.03636.x

Lee J, Lee S, Young JPW (2008) Improved PCR primers for the detection and identification of arbuscular mycorrhizal fungi. FEMS Microbiol Ecol 65: 339–349. https://doi.org/10.1111/j.1574-6941.2008.00531.x

Sato K, Suyama Y, Saito M, Sugawara K (2005) A new primer for discrimination of arbuscular mycorrhizal fungi with polymerase chain reaction – denature gradient gel electrophoresis. Grassl Sci 51: 179–181. https://doi.org/10.1111/j.1744-697X.2005.00023.x

Simon L, Lalonde M, Bruns TD (1992) Specific amplification of 18S fungal ribosomal genes from VA endomycorrhizal fungi colonizing roots. Appl Environ Microbiol 58: 291–295. https://doi.org/10.1128/aem.58.1.291-295.1992

# Appendix EA3 – Bioinformatic analysis

## Pre-processing raw Illumina reads

The sequence datasets were processed using software tools implemented in the SEED ver. 2.0 platform (Větrovský et al. 2018), Mothur ver. 1.39.5 (Schloss et al. 2009) and PipeCraft ver. 1.0 platform (Anslan et al. 2017).

For G-AMF data obtained by paired-end 2 × 300 bp sequencing (to describe G‑AMF communities), paired-end reads were assembled using FLASH ver. 1.2.11 (Magoč and Salzberg 2011) with the following settings: *minOverlap* = 10 bp, *mismatchRatio* = 0.3, *average read length* = 550, *SD* = 35. Quality filtering was performed using VSEARCH ver. 1.11.1 (Rognes et al. 2016) with the following settings: *truncqual* = 6, *maxee* = off, *maxee_rate* = 0.01, *minlen* = 450, *maxns* = 0. The resulting sequences were demultiplexed using SEED ver. 2.0 with no barcode mismatch allowed and primer mismatch set to 1, yielding 4,822,518 sequences spanning the complete amplicon length. Barcode and primer sequence were trimmed, and potentially chimeric sequences were removed using Uparse’ Usearch algorithm (Edgar 2013), yielding 4,534,943 pre-processed sequences.

For the G-AMF and M-FRE data obtained by paired-end 2 × 250 bp sequencing (primer pair AMV4.5NF–AMDGR; G-AMF sequences amplified by these primers were used only to calculate G-AMF/M-FRE ratios), paired-end reads were assembled using following settings: *minOverlap* = 15 bp, *mismatchRatio* = 0.25, *maxOverlap* = 250 bp. Quality filtering and demultiplexing was performed as above, except that the *minlen* parameter was set to 150, and primer mismatch was set to 3, yielding 9,264,633 sequences spanning the complete amplicon length. Barcode and primer sequences were trimmed, and potentially chimeric sequences were removed as described above, yielding 8,889,346 pre-processed sequences.

## Identification of G-AMF virtual taxa (VTX)

Typically, up to 1,000 of pre-processed sequences obtained by paired-end 2 × 300 bp sequencing were initially picked at random from each sample (additional sequences were added for samples with fewer resulting AMF sequences) and the dataset (1,097,593 sequences) was identified by BLAST against existing AMF sequences, using the ssu pipeline (Vasar et al. 2017) and extended MaarjAM database (6886 sequences, representing 384 VTX; Öpik et al. 2010; supplemented with a total of 40 sequences of two novel *Glomus* taxa not available in MaarjAM, but recorded in our previous data from the site – Šmilauer et al. 2021a,b). The following criteria were required for a match: sequence similarity ≥ 90%; alignment length ≥ 95% (i.e. not differing from the length of the shorter of the query and subject sequences by > 5%); and Blast e-value < 1e‑50. Up to 400 sequences matching the database were picked at random from each sample.

Sequences with similarity to closest available AMF sequence ≥ 90% but < 97% (5,050 sequences) were considered putative novel AMF taxa and were clustered using Usearch with a 97% similarity threshold. Only clusters containing more than 100 sequences were retained.

Twenty sequences of each cluster were selected at random and aligned with type sequences from the MaarjAM database using MAFFT ver. 7 (Katoh et al. 2017) with default settings and trimmed to the length of the Illumina sequences. A neighbor-joining tree was constructed using TOPALi ver. 2.5 (Milne et al. 2009) with default settings and 500 bootstrap replicates. Only the clusters which formed well-supported (bootstrap values ≥ 75) monophyletic clades were accepted. No novel VTX were detected using these criteria. Therefore, only sequences with similarity ≥ 97% to available sequences of AMF from the extended version of the MaarjAM database and ≥ 95% alignment length were retained in the dataset (257,581 sequences). Up to 400 of such target sequences were picked at random for each sample, and the resulting dataset of a total of 129,930 sequences was summarized using ssu pipeline. The VTX with read frequency < 0.5% per sample were removed from all samples prior to statistical analysis.

## Identification of M-FRE OTU

We decided to identify operational taxonomic units (OTU) of M-FRE by following Albornoz et al. (2021), who considered all OTU assigned to the order Endogonales as putative symbionts and thus Mucoromycotina fine root endophytes (M-FRE). As there is yet no database of verified M‑FRE sequences, we build our custom Endogonales database using (1) published sequences to which we refer as *expected symbiotic M-FRE* hereafter (50 Endogonales sequences published by Bidartondo et al. 2011; Orchard et al. 2017; Rimington et al. 2015), (2) Endogonales sequences available in SILVA database (Quast et al. 2013, accessed 11/7/2024; 185 sequences spanning at least the sequenced amplicon and not yet present in the dataset of expected symbiotic M-FRE), and (3) sequences of three novel Endogonales clusters deteceted in our previous study (Šmilauer et al. 2025; 60 sequences). In total 295 database sequences were clustered into 74 OTU at minimal 97% similarity nucleotide identity, using the average neighbor algorithm in Mothur (dist.seqs with *calc*=eachgap and *countends*=F options; cluster.classic with *method*=average and *cutoff*=0.03).

The dataset of 8,889,346 pre-processed AMV4.5NF‑AMDGR amplicon sequences was identified by BLAST searches against the above described Endogonales database using ssu pipeline. The following matching criteria were used: sequence identity ≥ 90%; alignment length ≥ 95%; and Blast e-value < 1e-50. Up to 400 sequences matching the database were picked at random from each sample for OTU inference.

Sequences with similarity ≥ 90% and < 97% to reference sequences from databases (45,900 sequences) were considered as being affiliated to putatively novel Endogonales taxa, and were clustered using Usearch with at least 97% nucleotide identity. Clusters containing more than 100 sequences were retained (42 clusters) and the most abundant sequence of each cluster assigned taxonomically following the SILVA taxonomy and using the SILVA alignment classifier, available as SILVA Alignment, Classification and Tree (ACT) service at <https://www.arb-silva.de/aligner/>. Only clusters whose most abundant sequence was at least ≥ 90% identical to a reference sequence of the Endogonales (33 clusters) were retained with 20 representative sequences selected at random. All these sequences were aligned together with the reference sequences of our newly created database of Endogonales (74 OTU) using MAFFT ver. 7 (Katoh et al. 2017) with default settings. A neighbor joining tree was constructed using TOPALi ver. 2.5 (Milne et al. 2009) with default settings and 500 bootstrap replicates. Only clusters which formed well-supported (bootstrap values ≥ 75) monophyletic clades were accepted. Three novel Endogonales clusters were detected using these criteria (CL0006, CL0016, CL0027) and 20 sequences of each of these clusters were used to extend our Endogonales database.

The final Endogonales database (74 Endogonales OTU + 3 novel Endogonales clusters) was used for the final taxonomic assignment of the 8,889,346 pre-processed sequences using BLAST. The following criteria were required for a match: at ≥ 97% sequence identity and alignment ≥ 95%; and Blast e-value < 1e-50. 382,303 sequences matched the extended Endogonales database among which we picked up to 400 sequences per sample at random. The resulting 104,984 picked sequences were summarised using the ssu pipeline. Only OTU with read frequency at least 0.5% per sample were retained for statistical analysis.

## Calculation of G-AMF/M-FRE ratio

We used the results of paired-end 2 × 250 bp sequencing to estimate this ratio. For M‑FRE counts, we used 382,303 sequences assigned to Endogonales. For G-AMF, the sequences of M‑FRE were removed from the complete dataset of 8,889,346 pre-processed sequences. The remaining 8,507,043 sequences were BLAST-searched against the extended MaarjAM database as described earlier, yielding a total of 3,403,645 target G-AMF sequences. The sequences of both types then were assigned to individual samples and G-AMF/M‑FRE ratio was calculated for each sample.

## References

Albornoz FE, Orchard S, Standish RJ, Dickie IA, Bending GD, Hilton S, Lardner T, Foster KJ, Gleeson DB, Bougoure J, Barbetti MJ, You MP, Ryan MH (2021) Evidence for niche differentiation in the environmental responses of co-occurring mucoromycotinian fine root endophytes and glomeromycotinian arbuscular mycorrhizal fungi. Microb Ecol 81: 864–873. https://doi.org/10.1007/s00248-020-01628-0

Anslan S, Bahram M, Hiiesalu I, Tedersoo L (2017) PipeCraft: Flexible open-source toolkit for bioinformatics analysis of custom high-throughput amplicon sequencing data. Mol Ecol Resour 17: e234–e240. https://doi.org/10.1111/1755-0998.12692

Bidartondo MI, Read DJ, Trappe JM, Merckx V, Ligrone R, Duckett JG (2011) The dawn of symbiosis between plants and fungi. Biol Letters 7: 574–577. https://doi.org/10.1098/rsbl.2010.1203

Chang Y, Desiró A., Na H, Sandor L, Lipzen A, Clum A, Barry K, Grigoriev IV, Martin FM, Stajich JE, Smith ME, Bonito G, Spatafora JW (2019) Phylogenomics of Endogonaceae and evolution of mycorrhizas within Mucoromycota. New Phytol 222: 511–525. https://doi.org/10.1111/nph.15613

Edgar RC (2013) UPARSE: highly accurate OTU sequences from microbial amplicon reads. Nat Methods 10: 996–998. https://doi.org/10.1038/NMETH.2604

Katoh K, Rozewicki J, Yamada KD (2017) MAFFT online service: multiple sequence alignment, interactive sequence choice and visualization. Brief Bioinf 20: 1160–1166. URL <https://mafft.cbrc.jp/alignment/server/index.html>. https://doi.org/10.1093/bib/bbx108

Magoč T, Salzberg SL (2011) FLASH: fast length adjustment of short reads to improve genome assemblies. Bioinformatics 27: 2957–2963. https://doi.org/10.1093/bioinformatics/ btr507

Milne I, Lindner D, Bayer M, Husmeier D, McGuire G, Marshall DF, Wright F (2009) TOPALi v2: a rich graphical interface for evolutionary analyses of multiple alignments on HPC clusters and multi-core desktops. Bioinformatics 25: 126–127. https://doi.org/10.1093/bioinformatics/btn575

Öpik M, Vanatoa A, Vanatoa E, Moora M, Davison J, Kalwij JM, Reier U, Zobel M (2010) The online database MaarjAM reveals global and ecosystemic distribution patterns in arbuscular mycorrhizal fungi (Glomeromycota). New Phytol 188: 223–241. https://doi.org/10.1111/ j.1469-8137.2010.03334.x

Orchard S, Hilton S, Bending GD, Dickie IA, Standish RJ, Gleeson DB, Jeffery RP, Powell JR, Walker C, Bass D, Monk J, Simonin A, Ryan MH (2017) Fine endophytes (*Glomus tenue*) are related to Mucoromycotina, not Glomeromycota. New Phytol 213: 481–486. https://doi.org/10.1111/nph.14268

Quast C, Pruesse E, Yilmaz P, Gerken J, Schweer T, Yarza P, Peplies J, Glöckner FO (2013) The SILVA ribosomal RNA gene database project: improved data processing and web-based tools. Nucleic Acids Res 41(D1): D590–D596. https://doi.org/10.1093/nar/gks1219

Rimington WR, Pressel S, Duckett JG, Bidartondo MI (2015) Fungal associations of basal vascular plants: reopening a closed book? New Phytol 205: 1394–1398. https://doi.org/10.1111/nph.13221

Rognes T, Flouri T, Nichols B, Quince C, Mahé F (2016) VSEARCH: a versatile open source tool for metagenomics. Peer J 4: e2584. https://doi.org/10.7717/peerj.2584

Schloss PD, Westcott SL, Ryabin T, Hall JR, Hartmann M, Hollister EB, Lesniewski RA, Oakley BB, Parks DH, Robinson CJ, Sahl JW, Stres B, Thallinger GG, Van Horn DJ, Weber CF (2009) Introducing Mothur: Open source, platform-independent, community-supported software for describing and comparing microbial communities. Appl Env Microbiol 75: 7537–7541. https://doi.org/10.1128/AEM.01541-09

Šmilauer P, Košnar J, Kotilínek M, Pecháčková S, Šmilauerová M (2021a) Host age and surrounding vegetation affect the community and colonization rates of arbuscular mycorrhizal fungi in a temperate grassland. New Phytol 232: 290–302. https://doi.org/10.1111/nph.17550

Šmilauer P, Šmilauerová M, Kotilínek M, Košnar J (2021b) Arbuscular mycorrhizal fungal communities of forbs and C3 grasses respond differently to cultivation and elevated nutrients. Mycorrhiza 31: 455–470. https://doi.org/10.1007/s00572-021-01036-3

Šmilauer P, Šmilauerová M, Košnar J (2025) Functional group of grassland plants affects their fungal symbionts more than long-term fertilisation in a field experiment. Applied Soil Ecology 213: 106288. https://doi.org/10.1016/j.apsoil.2025.106288

Vasar M, Andreson R, Davison J, Jairus T, Moora M, Remm M, Young JPW, Zobel M, Öpik M (2017) Increased sequencing depth does not increase captured diversity of arbuscular mycorrhizal fungi. Mycorrhiza 27: 761–773. https://doi.org/10.1007/s00572-017-0791-y

Větrovský T, Baldrian P, Morais D (2018) SEED 2: A user-friendly platform for amplicon high-throughput sequencing data analyses. Bioinformatics 34: 2292–2294. https://doi.org/ 10.1093/bioinformatics/bty071

# Appendix EA4 – Models of statistical analysis

## Multivariate models

All multivariate analyses used a constrained linear ordination method of redundancy analysis (RDA, Legendre and Legendre 2012) in its partial form (i.e. with covariates). Permutations tests reflected the experimental design by using covariates and, where appropriate, the design-based permutation type (Ter Braak and Šmilauer 2018). As we tried to achieve an equal number of 400 NGS reads for each analysed case, lower number of reads available for a case was reflected in a reduced case weight, defined as N_reads_ / 400. All data tables containing read counts were ln(x+1)–transformed and Hellinger’ standardisation (Legendre and Legendre 2012) was applied to all cases. Presented amount of explained variation is the adjusted coefficient of determination and it uses the full community variation (of relative proportions) as 100%, so it does not exclude the variation explained by covariates.

Multivariate analyses reflected the hierarchical design, which was based on complete randomised blocks with four plot types (differing in fertilisation) repeated (with two replicates) in each of 12 sites, and with four or five plant species planted within each plot. The resulting dataset was not completely balanced, as (a) the *Betonica officinalis* seedlings were planted only in four sites, and (b) not all planted species survived in each plot. Site identity was used as a covariate defining permutation blocks, except when testing among-site variation (namely the effects of N and P availability). The host species identity was also used as a covariate defining (together with sites) the permutation blocks when examining the effects of nutrient addition, but not when examining the host species effects.

When visualising ordination results with ordination diagrams, we used biplot diagrams with the arrows displayed for VTX or OTU selected by their important relationship to explanatory variables, as indicated by t-value biplots for the ordination model (Ter Braak and Šmilauer 2018). To interpret ordination biplots, individual taxa (shown as arrows) can be related to predictor variables depending on their presentation in the biplot. When shown as symbols (for a categorical predictor, with the symbols representing individual categories), they can be perpendicularly projected onto a (prolonged) taxon arrow to deduce the average taxon abundance in samples of a particular category. The taxon arrow points into the direction of increasing abundance values and the average abundance is located at zero point. For a quantitative predictor (represented in the diagram by an arrow), the angle between predictor and taxon arrows approximates their correlation (estimated as the cosine of the angle), When both arrows point in the same direction or span a narrow angle, a positive correlation is predicted; when they point into opposite directions or span a wide angle, a negative correlation is predicted. The angle between arrows of two taxa corresponds to their correlation.

## Regression models

When testing hypotheses about univariate response variables, we used linear mixed-effect models (LMMs) with log-transformed response, fitted using the *lme4* package (Bates et al. 2015). To test hypotheses about fixed effects, we used likelihood ratio test examining the change in model log‑likelihood after dropping the tested term from the model. We used similar approach to estimate the variation explained by a model term as the difference between marginal coefficients of determination estimated for models with and without the concerned term, utilising the *MuMIn* package (Bartoň 2024). As all the response variables were log-transformed, we calculated the effect sizes by exponentiating estimated regression coefficient and presenting this as a proportional (multiplicative) change due to a change in the predictor value. We used Hill’s N2 diversity index (Legendre and Legendre 2012) to measure α‑diversity of fungal communities, and we complemented it with log-transformed VTX / OTU richness.

Our blue-print LMM included fixed main effects of host species identity, N addition, and P addition, as well as the N and P interaction. Site and plot identities represented random effects. We used the weights for individual observations, representing either the proportion of read count out of the target 400 reads (as in multivariate analyses) or – for colonisation levels estimated by microscopy – the average visual quality of stained root samples. The LMMs were used to estimate the significance, effect size and explained variation for host species, N:P interaction, and among-site variation by dropping respective terms from the model. Afterwards, the N:P interaction was dropped for each model (it was not significant in any of the estimated models) and the main effects of N and P were examined. Additionally, we explored possible differences in fungal community response to fertilisation among the host plant species by testing the interaction between N or P and the host species identity. When examining the effect of N or P availability (see Appendix EA1 concerning their estimation), these predictors were added (separately) to a blue-print model described above. To test our H3 hypothesis, we extended a model including P availability by its interaction with N addition.

## References

Bartoň K (2024) MuMIn: multi-model inference. R package version 1.48.4, <https://cran.r-project.org/package=MuMIn>

Bates D, Mächler M, Bolker B, Walker S (2015) Fitting linear mixed-effects models using lme4. J Stat Soft 67: 1–48. <https://doi.org/10.18637/jss.v067/i01>

Legendre P, Legendre L (2012) Numerical Ecology. 3rd English edition. Amsterdam, 950 pp., Elsevier, Amsterdam, The Netherlands

Ter Braak CJF, Šmilauer P (2018) Canoco Reference Manual and User’s Guide: Software for Ordination (version 5.1). Microcomputer Power, Ithaca, USA. 536 pp.

# Appendix EA5 – Overview of G-AMF VTX and M-FRE OTU

The quality filtered dataset of 18S rDNA sequences amplified with NS31 – AML2 primers yielded 107 G-AMF VTXs (see Table EA2 for their list), represented by 126,048 reads in the analysed data. The most VTX-rich was the family Glomeraceae (71 VTX), followed by the Acaulosporaceae (9 VTX), Claroideoglomeraceae (8 VTX), Archaeosporaceae (6 VTX), Gigasporaceae (6 VTX), Paraglomeraceae (4 VTX), and Diversisporaceae (3 VTX) families. Proportions of DNA sequences belonging to individual G-AMF families are visualised for individual host plant species and for the fertilisation treatments in Fig. EA2. The participation of G‑AMF families is similar across all compared host species and also among experimental treatments, although N addition seems to increase compositional evenness at the family level.

**Table EA2** – The list of G-AMF VTXs identified in the dataset based on NS31 – AML2 primers. First column shows the original code in MaarjAM database (Öpik et al. 2010) – except the first two entries, which represent novel VTXs. Second column displays shorter code version used in this paper, *Family* column assigns individual VTXs to G-AMF families, *Genbank code* provides reference to Genbank deposition. *Proportion* column displays the percentage of sequence reads assigned to a particular VTX in the G-AMF dataset. VTXs with value *yes* in *Cultivation* column have at least one record in the MaarjAM database with the “Source” field specified as “cultured spores”. The *Morphospecies* column provides information of the relationship with described morphotaxa, based on the data available in the MaarjAM database.

| **VTX code (MaarjAM)** | **Code paper** | **Family** | **Genbank code** | **Proportion**nn | **Cultivation** | **Morphospecies** |
| --- | --- | --- | --- | --- | --- | --- |
| CL004 | CL04 | Glomeraceae | PX244395 | 0.06 | no |  |
| CL006 | CL06 | Glomeraceae | PX244396 | 0.07 | no |  |
| VTX00004 | t004 | Archaeosporaceae | PX244397 | 0.93 | no |  |
| VTX00005 | t005 | Archaeosporaceae | PX244398 | 2.47 | no |  |
| VTX00008 | t008 | Archaeosporaceae | PX244399 | 0.01 | no |  |
| VTX00010 | t010 | Acaulosporaceae | PX244400 | 0.38 | no |  |
| VTX00012 | t012 | Acaulosporaceae | PX244401 | <0.01 | no |  |
| VTX00013 | t013 | Acaulosporaceae | PX244402 | 0.04 | no |  |
| VTX00015 | t015 | Acaulosporaceae | PX244403 | 0.04 | no |  |
| VTX00028 | t028 | Acaulosporaceae | PX244404 | 0.01 | yes | A. longula, A. rugosa |
| VTX00030 | t030 | Acaulosporaceae | PX244405 | 7.14 | yes | A. scrobiculata |
| VTX00039 | t039 | Gigasporaceae | PX244406 | 0.01 | yes | G. decipiens, G. ,argarota |
| VTX00049 | t049 | Gigasporaceae | PX244407 | 0.20 | yes | Scutellospora dipurpurescens, S. calospora |
| VTX00052 | t052 | Gigasporaceae | PX244408 | 0.27 | yes | Scutellospora calospora, S. aurigloba |
| VTX00055 | t055 | Claroideoglomeraceae | PX244409 | <0.01 | no |  |
| VTX00056 | t056 | Claroideoglomeraceae | PX244410 | 0.37 | no |  |
| VTX00057 | t057 | Claroideoglomeraceae | PX244411 | 1.16 | no |  |
| VTX00061 | t061 | Diversisporaceae | PX244412 | <0.01 | yes | D. epigaea, D. spurca, Glomus versiforme |
| VTX00062 | t062 | Diversisporaceae | PX244413 | 0.01 | no |  |
| VTX00063 | t063 | Glomeraceae | PX244414 | 0.01 | yes | Glomus viscosum |
| VTX00064 | t064 | Glomeraceae | PX244415 | 0.02 | yes | G. constrictum |
| VTX00072 | t072 | Glomeraceae | PX244416 | 1.14 | no |  |
| VTX00074 | t074 | Glomeraceae | PX244417 | 19.32 | yes |  |
| VTX00078 | t078 | Glomeraceae | PX244418 | 0.14 | no |  |
| VTX00092 | t092 | Glomeraceae | PX244419 | <0.01 | no |  |
| VTX00096 | t096 | Glomeraceae | PX244420 | 0.03 | no |  |
| VTX00108 | t108 | Glomeraceae | PX244421 | 9.15 | no |  |
| VTX00113 | t113 | Glomeraceae | PX244422 | 9.58 | yes | Rhizophagus irregularis |
| VTX00114 | t114 | Glomeraceae | PX244423 | 0.17 | yes | Rhizophagus irregularis |
| VTX00115 | t115 | Glomeraceae | PX244424 | 3.23 | yes | Rhizophagus irregularis |
| VTX00117 | t117 | Glomeraceae | PX244425 | 0.30 | no |  |
| VTX00118 | t118 | Glomeraceae | PX244426 | 0.01 | no |  |
| VTX00122 | t122 | Glomeraceae | PX244427 | 0.33 | no |  |
| VTX00125 | t125 | Glomeraceae | PX244428 | 0.02 | no |  |
| VTX00128 | t128 | Glomeraceae | PX244429 | <0.01 | no |  |
| VTX00129 | t129 | Glomeraceae | PX244430 | 0.13 | no |  |
| VTX00130 | t130 | Glomeraceae | PX244431 | 0.07 | no |  |
| VTX00135 | t135 | Glomeraceae | PX244432 | 0.19 | no |  |
| VTX00137 | t137 | Glomeraceae | PX244433 | 0.09 | no |  |
| VTX00142 | t142 | Glomeraceae | PX244434 | <0.01 | no |  |
| VTX00143 | t143 | Glomeraceae | PX244435 | 0.21 | no |  |
| VTX00149 | t149 | Glomeraceae | PX244436 | 0.62 | no |  |
| VTX00151 | t151 | Glomeraceae | PX244437 | <0.01 | no |  |
| VTX00153 | t153 | Glomeraceae | PX244438 | 0.20 | no |  |
| VTX00155 | t155 | Glomeraceae | PX244439 | <0.01 | no | G. iranicum ? |
| VTX00159 | t159 | Glomeraceae | PX244440 | 0.11 | no |  |
| VTX00160 | t160 | Glomeraceae | PX244441 | 0.02 | no |  |
| VTX00163 | t163 | Glomeraceae | PX244442 | 3.92 | no |  |
| VTX00166 | t166 | Glomeraceae | PX244443 | 4.21 | no |  |
| VTX00167 | t167 | Glomeraceae | PX244444 | 0.01 | no |  |
| VTX00172 | t172 | Glomeraceae | PX244445 | 0.01 | no |  |
| VTX00184 | t184 | Glomeraceae | PX244446 | 0.01 | no |  |
| VTX00186 | t186 | Glomeraceae | PX244447 | 0.31 | no |  |
| VTX00191 | t191 | Glomeraceae | PX244448 | 1.71 | no |  |
| VTX00193 | t193 | Claroideoglomeraceae | PX244449 | 0.49 | yes | C.claroideum, C. lamellosum, C.luteum |
| VTX00194 | t194 | Glomeraceae | PX244450 | 0.48 | no |  |
| VTX00199 | t199 | Glomeraceae | PX244451 | 0.07 | yes | G. hoi |
| VTX00202 | t202 | Glomeraceae | PX244452 | <0.01 | no |  |
| VTX00212 | t212 | Glomeraceae | PX244453 | 1.31 | no |  |
| VTX00213 | t213 | Glomeraceae | PX244454 | <0.01 | no |  |
| VTX00214 | t214 | Glomeraceae | PX244455 | 0.03 | no |  |
| VTX00216 | t216 | Glomeraceae | PX244456 | 0.21 | no |  |
| VTX00219 | t219 | Glomeraceae | PX244457 | 9.16 | no |  |
| VTX00222 | t222 | Glomeraceae | PX244458 | 0.07 | no | G. indicum ? |
| VTX00223 | t223 | Glomeraceae | PX244459 | 0.62 | no |  |
| VTX00225 | t225 | Claroideoglomeraceae | PX244460 | 1.48 | no |  |
| VTX00228 | t228 | Acaulosporaceae | PX244461 | 0.97 | no |  |
| VTX00231 | t231 | Acaulosporaceae | PX244462 | 0.98 | yes |  |
| VTX00233 | t233 | Glomeraceae | PX244463 | 0.56 | no |  |
| VTX00234 | t234 | Glomeraceae | PX244464 | <0.01 | no |  |
| VTX00239 | t239 | Paraglomeraceae | PX244465 | 0.08 | yes | P. brasilianum |
| VTX00245 | t245 | Archaeosporaceae | PX244466 | 3.71 | yes | A.trappei, A.schenckii |
| VTX00247 | t247 | Glomeraceae | PX244467 | 0.48 | no |  |
| VTX00254 | t254 | Gigasporaceae | PX244468 | <0.01 | yes | Scutellospora spinosissima, Cetraspora nodosa |
| VTX00255 | t255 | Gigasporaceae | PX244469 | 0.01 | yes | Scutellospora heterogama, S. reticulata |
| VTX00256 | t256 | Glomeraceae | PX244470 | 0.37 | no |  |
| VTX00276 | t276 | Claroideoglomeraceae | PX244471 | 0.45 | no |  |
| VTX00278 | t278 | Claroideoglomeraceae | PX244472 | 0.02 | no |  |
| VTX00281 | t281 | Paraglomeraceae | PX244473 | 1.27 | yes | P. laccatum |
| VTX00305 | t305 | Glomeraceae | PX244474 | 0.02 | no |  |
| VTX00309 | t309 | Glomeraceae | PX244475 | <0.01 | no |  |
| VTX00312 | t312 | Glomeraceae | PX244476 | <0.01 | no |  |
| VTX00315 | t315 | Glomeraceae | PX244477 | 1.40 | no |  |
| VTX00318 | t318 | Gigasporaceae | PX244478 | <0.01 | no |  |
| VTX00323 | t323 | Glomeraceae | PX244479 | <0.01 | no |  |
| VTX00324 | t324 | Glomeraceae | PX244480 | <0.01 | no |  |
| VTX00325 | t325 | Glomeraceae | PX244481 | 2.05 | no | Rhizophagus sp. |
| VTX00326 | t326 | Glomeraceae | PX244482 | 0.01 | no |  |
| VTX00337 | t337 | Paraglomeraceae | PX244483 | 0.61 | no |  |
| VTX00338 | t338 | Archaeosporaceae | PX244484 | 0.06 | no |  |
| VTX00340 | t340 | Claroideoglomeraceae | PX244485 | 0.33 | no |  |
| VTX00342 | t342 | Glomeraceae | PX244486 | 0.16 | no |  |
| VTX00344 | t344 | Glomeraceae | PX244487 | 0.04 | no |  |
| VTX00345 | t345 | Glomeraceae | PX244488 | 0.01 | no |  |
| VTX00349 | t349 | Paraglomeraceae | PX244489 | 0.04 | no |  |
| VTX00366 | t366 | Glomeraceae | PX244490 | 0.15 | no |  |
| VTX00371 | t371 | Glomeraceae | PX244491 | 0.18 | no |  |
| VTX00373 | t373 | Glomeraceae | PX244492 | 0.01 | no |  |
| VTX00379 | t379 | Acaulosporaceae | PX244493 | 0.11 | no |  |
| VTX00380 | t380 | Diversisporaceae | PX244494 | <0.01 | no |  |
| VTX00386 | t386 | Glomeraceae | PX244495 | 0.02 | no |  |
| VTX00407 | t407 | Glomeraceae | PX244496 | 0.04 | no |  |
| VTX00411 | t411 | Glomeraceae | PX244497 | 0.01 | no |  |
| VTX00417 | t417 | Glomeraceae | PX244498 | 0.67 | no |  |
| VTX00423 | t423 | Glomeraceae | PX244499 | 0.06 | no |  |
| VTX00431 | t431 | Glomeraceae | PX244500 | 2.77 | no |  |
| VTX00456 | t456 | Archaeosporaceae | PX244501 | <0.01 | no |  |


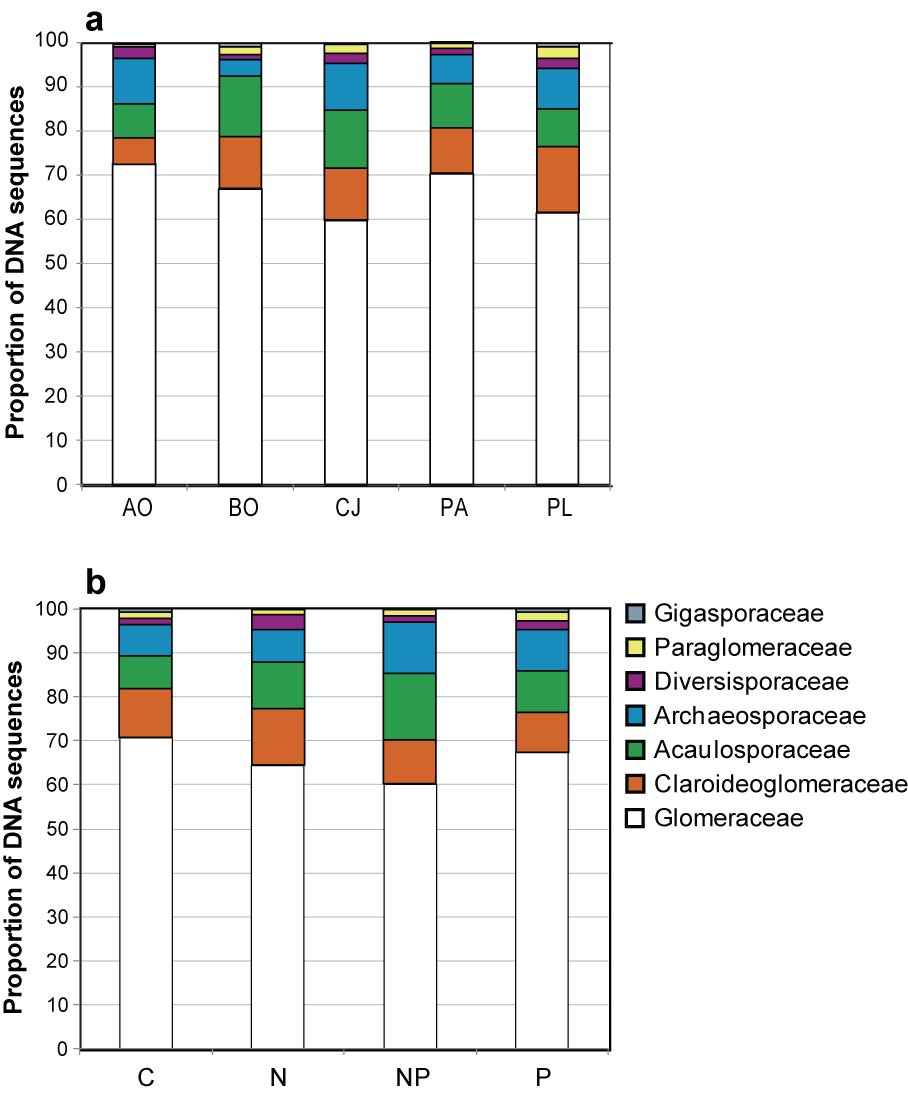


**Fig. EA2** – The proportion of individual G-AMF families (based on DNA sequence counts) for individual host species (**a**) and for fertilisation treatments (**b**). Species acronyms: *AO* – *Anthoxanthum odoratum*, *BO* – *Betonica officinalis*, *CJ* – *Centaurea jacea*, *PA* – *Poa angustifolia*, *PL* – *Plantago lanceolata*; Fertilisation treatment acronyms: *C* – control plots, *N* – nitrogen added, *NP* – nitrogen and phosphorus added, *P* – phosphorus added.

The quality filtered dataset of 18S rDNA sequences amplified with the PCR primers AMV4.5NF and AMDGR contained 19 OTUs of Endogonales order (see Table EA3 for their enumeration), represented by 104,588 reads in the analysed data table. Five OTUs were affiliated to sequences previously recognised as M-FRE in plant symbionts (two OTUs in Bidartondo et al. 2011 and two OTUs in Orchard et al. 2017, one OTU in both references). The most abundant OTU004, representing 59.9% of NGS reads, includes sequences identified as *OTU0* in Orchard et al. 2017 (where it was also found as the most abundant OTU with 44.6% of reads) and as 8388 OTU in Bidartondo et al. (2011). The second most abundant OTU012 (15.3% of NGS reads) contained no known M-FRE sequences and thus seems to represent a new symbiont, similarly to OTU *CL0016* represented by 12.9% of total DNA sequences. Remaining OTU were all represented by less than 4% of the total number of sequences.

**Table EA3** – The list of M‑FRE OTUs identified in the dataset based on AMV4.5NF–AMDGR primers. First column shows the codes used in this paper. *Family/Genus* column provides approximate taxonomic position according to Tedersoo et al. 2024 (the most abundant sequence of each OTU cluster was blast-searched against available Endogonomycetes SSU sequences deposited in Eukaryome v1.8 – accessed 3/10/2024; *n.a.* indicates the absence of classification at the given level in the most similar reference sequence). The *Genbank code* column gives the deposit code of the most abundant sequence detected in our study. The *Source* lists the references that included DNA sequences of Endogonales which clustered into given OTU. *Frequency* column shows the percentage of total OTU reads out of the whole M-FRE dataset.

| **OTU code** | **Family** | **Genus** | **Genbank code** | **Source** | **Frequency  [%]** |
| --- | --- | --- | --- | --- | --- |
| CL0006 | Planticonsortiaceae | n.a. | PX244502 | this study | 1.60 |
| CL0016 | Planticonsortiaceae | n.a. | PX244503 | this study | 12.88 |
| CL0025 | Planticonsortiaceae | n.a. | PX244504 | Šmilauer et al 2025 | 0.82 |
| CL0027 | Planticonsortiaceae | n.a. | PX244505 | this study | 0.56 |
| CL0028 | Planticonsortiaceae | n.a. | PX244506 | Šmilauer et al 2025 | 2.71 |
| CL0038 | Planticonsortiaceae | n.a. | PX244507 | Šmilauer et al 2025 | 0.19 |
| OTU004 | Planticonsortiaceae | n. a. | PX244508 | Bidartondo et al 2011, Orchard et al 2017 | 59.87 |
| OTU005 | n.a. | n. a. (Endogonales) | PX244509 | Quast et al 2013 | <0.01 |
| OTU012 | Planticonsortiaceae | n.a. | PX244510 | Quast et al 2013 | 15.32 |
| OTU013 | n.a. | n.a. (Densosporales) | PX244511 | Bidartondo et al 2011 | 0.19 |
| OTU016 | Planticonsortiaceae | Planticonsortium | PX244512 | Bidartondo et al 2011 | 1.15 |
| OTU017 | Planticonsortiaceae | Planticonsortium | PX244513 | Orchard et al 2017 | 3.18 |
| OTU023 | n.a. | n.a. (Densosporales) | PX244514 | Quast et al 2013 | 0.04 |
| OTU024 | n.a. | n.a. (Densosporales) | PX244515 | Quast et al 2013 | 0.04 |
| OTU026 | Endogonaceae | Endogone | PX244516 | Orchard et al 2017 | 0.01 |
| OTU033 | Planticonsortiaceae | n.a. | PX244517 | Quast et al 2013 | 0.02 |
| OTU034 | n.a. | n.a. (Densosporales) | PX244518 | Quast et al 2013 | 0.04 |
| OTU045 | n. a. | n.a. (Densosporales) | PX244519 | Quast et al 2013 | <0.01 |
| OTU069 | Planticonsortiaceae | n.a. | PX244520 | Quast et al 2013 | 1.39 |

## References

Bidartondo MI, Read DJ, Trappe JM, Merckx V, Ligrone R, Duckett JG (2011) The dawn of symbiosis between plants and fungi. Biol Letters 7: 574–577. https://doi.org/10.1098/rsbl.2010.1203

Öpik M, Vanatoa A, Vanatoa E, Moora M, Davison J, Kalwij JM, Reier U, Zobel M (2010) The online database MaarjAM reveals global and ecosystemic distribution patterns in arbuscular mycorrhizal fungi (Glomeromycota). New Phytol 188: 223–241. https://doi.org/10.1111/ j.1469-8137.2010.03334.x

Orchard S, Hilton S, Bending GD, Dickie IA, Standish RJ, Gleeson DB, Jeffery RP, Powell JR, Walker C, Bass D, Monk J, Simonin A, Ryan MH (2017) Fine endophytes (*Glomus tenue*) are related to Mucoromycotina, not Glomeromycota. New Phytol 213: 481–486. https://doi.org/10.1111/nph.14268

Quast C, Pruesse E, Yilmaz P, Gerken J, Schweer T, Yarza P, Peplies J, Glöckner FO (2013) The SILVA ribosomal RNA gene database project: improved data processing and web-based tools. Nucleic Acids Res 41(D1): D590–D596. https://doi.org/10.1093/nar/gks1219

Šmilauer P, Šmilauerová M, Košnar J (2025) Functional group of grassland plants affects their fungal symbionts more than long-term fertilisation in a field experiment. Applied Soil Ecology 213: 106288. https://doi.org/10.1016/j.apsoil.2025.106288

Tedersoo L, Magurno F, Alkahtani S, Mikryukov V (2024) Phylogenetic classification of arbuscular mycorrhizal fungi: new species and higher-ranking taxa in Glomeromycota and Mucoromycota (class Endogonomycetes). MycoKeys 107: 273–325. https://doi.org/10.3897/mycokeys.107.125549

# Appendix EA6 – Soil properties, biomass of seedlings and plant community

We evaluated the effects of fertilisation treatment and differences among sampling sites using linear mixed-effect models (using *nlme* package, Pinheiro et al 2023) with a fixed effect of treatment (extended, in the case of seedling aboveground biomass, by a fixed effect of plant species identity) and a random effect of site identity. All response variables (concentrations, biomass) were log-transformed except pH. We tested the treatment effect using an F-test and the variation of response variable among the sites by comparing the fitted model with a linear model without the random effect of site using the likelihood-ratio (χ^2^) test. The nature of treatment effect was examined by performing multiple comparisons among treatment levels (Control, N, P, and N+P) using the *multcomp* package (Hothorn et al. 2008). We estimated the amount of explained variation both by the fixed effect of treatment and the random effect of site using the *MuMIn* package (Bartoň 2024).

Graphical summary of the responses of soil and aboveground biomass characteristics to fertilisation is based on the partial redundancy analysis (RDA, Legendre and Legendre 2012 ), using fertilisation treatment as a categorical predictor and site identity as a covariate.

## Soil properties

Observed values of soil properties, measured in 2024 (one year after the seedling experiment with continuing fertilisation treatments) are summarised in Table EA4, site characteristics are computed using control, non-fertilised plots. The N addition (with or without P) increased available N in soil from 10.7 mg / kg of DW soil in control and P plots to 21.5 mg / kg in N and N+P plots. Similarly the P addition increased the inorganic P concentration from 28.6 mg / kg of DW soil in control and N plots to 51.6 mg / kg in P and N+P plots. N-only addition increased (by 187%) and P-only addition decreased (by 44%) the N / P ratio of available forms. N+P addition increased the ratio by 23%.

**Table EA4** – Summary of fertilisation-based differences and among-site variation of soil characteristics, measured in spring 2024. In the *Fertilisation effects* part, arithmetic averages are shown ± standard error. *Inorganic N* and *Inorganic P* show concentration (mg/kg of dry soil) of N in NO_3_^-^ and NH_4_^+^ ions, and of P in PO_4_^3-^ ions, respectively. *Total N* represents N percentage in soil dry weight. In the *Summary of site averages* part, summary statistics are presented for average characteristic values computed for individual sites using control (non-fertilised) plots.

|  |  | **pH** | **Inorganic**  **N** | **Inorganic**  **P** | **Total N** |
| --- | --- | --- | --- | --- | --- |
| **Fertilisation effects** | **Control plots** | 5.41 ± 0.07 | 11.2 ± 1.4 | 31.1 ± 3.97 | 0.26 ± 0.012 |
|  | **N plots** | 5.48 ± 0.06 | 23.0 ± 3.1 | 26.0 ± 3.89 | 0.28 ± 0.011 |
|  | **P plots** | 5.43 ± 0.07 | 10.2 ± 0.9 | 53.0 ± 6.20 | 0.25 ± 0.011 |
|  | **N+P plots** | 5.37 ± 0.07 | 19.9 ± 1.9 | 50.1 ± 6.23 | 0.28 ± 0.012 |
| **Summary of site averages** | **Minimum** | 4.71 | 4.10 | 5.57 | 0.215 |
|  | **Lower quartile** | 5.17 | 7.57 | 18.07 | 0.239 |
|  | **Median** | 5.45 | 9.13 | 30.57 | 0.250 |
|  | **Average** | 5.41 | 11.24 | 31.10 | 0.264 |
|  | **Upper quartile** | 5.65 | 13.41 | 42.75 | 0.273 |
|  | **Maximum** | 5.96 | 23.96 | 60.42 | 0.400 |

Table EA5 summarises the results of soil data analyses. Fertilisation did not affect soil pH, but we found a large among-site variation in pH values (with median of individual sites ranging from 4.7 to 5.8). The N addition significantly increased available N in soil and the P addition significantly increased the inorganic P concentration. Combined addition of N+P did not significantly change the N/P ratio, but separate additions of N or P did, in the expected directions.

**Table EA5** – Analyses of the response of soil parameters (pH, N in NO_3_^-^ and NH_4_^+^ ions, P in PO_4_^3-^ ions, and inorganic N/P ratio) to fertilisation treatment and site identity. All F-statistics have 3, 81 degrees of freedom, all χ^2^-statistics have 1 degree of freedom. *n. s.* – non-significant, *n. a.* – not appropriate.

|  | **Fertilisation treatment effect** | | | **Among-site variation** | |
| --- | --- | --- | --- | --- | --- |
|  | **Test F p** | **Explained variation [%]** | **Effect description** | **Test χ^2^  p** | **Explained variation [%]** |
| **Soil pH** | 2.01 n. s. | 1.3 | n. a. | 100.6 < 0.001 | 77.4 |
| **Inorganic N** | 26.1 < 0.001 | 34.5 | N & N+P  > C & P | 20.82 < 0.001 | 23.5 |
| **Inorganic P** | 36.0  < 0.001 | 18.5 | P & N+P  > C & N | 106.3 < 0.001 | 65.2 |
| **Inorganic N / P ratio** | 54.6 < 0.001 | 24.3 | N > C & NP > N | 111.9 < 0.001 | 61.6 |

## Community biomass and its N and P content

Aboveground biomass and its nutrient contents are summarised in Table EA6, with the site characteristics computed using control, non-fertilised plots.

**Table EA6** – Summary of fertilisation-based differences and among-site variation of plant community‘ aboveground biomass and its content of N and P, measured in spring 2024. In the *Fertilisation effects* part, arithmetic averages are shown ± standard error. *Aboveground Biomass DW* shows the aboveground biomass (cut 1 cm aboveground) in g/m^2^. *Nitrogen* and *Phosphorus* show values as weight percentages. In the *Summary of site averages* part, summary statistics are presented for average characteristic values computed for individual sites using control (non-fertilised) plots.

|  |  | **Aboveground**  **Biomass DW** | **Nitrogen** | **Phosphorus** | **N/P Ratio** |
| --- | --- | --- | --- | --- | --- |
| **Fertilisation effects** | **Control plots** | 124.9 ± 16.3 | 2.15 ± 0.10 | 0.23 ± 0.01 | 9.79 ± 0.61 |
|  | **N plots** | 146.2 ± 12.1 | 2.69 ± 0.06 | 0.20 ± 0.01 | 14.59 ± 1.09 |
|  | **P plots** | 132.4 ± 11.2 | 2.00 ± 0.07 | 0.29 ± 0.01 | 7.00 ± 0.28 |
|  | **N+P plots** | 178.8 ± 17.0 | 2.47 ± 0.14 | 0.31 ± 0.01 | 8.16 ± 0.45 |
| **Summary of site averages** | **Minimum** | 60.8 | 1.69 | 0.145 | 6.46 |
|  | **Lower quartile** | 83.7 | 2.01 | 0.179 | 7.86 |
|  | **Median** | 117.2 | 2.04 | 0.255 | 8.61 |
|  | **Average** | 124.9 | 2.15 | 0.233 | 9.80 |
|  | **Upper quartile** | 136.4 | 2.21 | 0.261 | 12.52 |
|  | **Maximum** | 260.3 | 3.35 | 0.320 | 14.23 |

Table EA7 summarises the response of aboveground and belowground biomass, as well as their N and P concentrations and N/P ratio to fertilisation and the variation of those characteristics among the sites. The weight of aboveground biomass increased due to fertilisation, particularly when N and P addition were combined. We found no change of belowground biomass due to fertilisation, possibly because the sampled soil depth was *a priori* fixed. N concentration responded to N addition (also in a combination with P addition) for the aboveground biomass, but in belowground biomass it responded just to N-only treatment. P concentration increased due to P addition (with or without N being added) both for aboveground and belowground biomass. N/P ratio in biomass increased due to adding N (without P) and the two treatments including P led to a decrease of the ratio. P concentration varied among sites more than the N concentration or N/P ratio, both in above- and belowground biomass.

**Table EA7** – Analyses of the response of biomass weight, concentration of N and P and their N/P ratio to fertilisation treatment and site identity; aboveground and belowground biomass were analysed separately. All F‑statistics have 3, 81 degrees of freedom, all χ^2^-statistics have 1 degree of freedom. *n.s.* – non-significant, *n.a.* – not appropriate.

|  | **Fertilisation treatment effect** | | | **Among-site variation** | |
| --- | --- | --- | --- | --- | --- |
|  | **Test F p** | **Explained variation [%]** | **Effect description** | **Test χ^2^  p** | **Explained variation [%]** |
| **Aboveground biomass** | 5.18 0.003 | 9.9 | N+P > N > P & C | 18.0 < 0.001 | 29.8 |
| **Belowground biomass** | 0.06 n. s. | 0.1 | n. a. | 16.7 < 0.001 | 31.6 |
| **N concentr. aboveground** | 13.7 < 0.001 | 28.8 | N & N+P >  P & C | 1.09 n. s. | 4.7 |
| **P concentr. aboveground** | 44.0 < 0.001 | 32.9 | P & N+P > C > N | 63.1 < 0.001 | 43.5 |
| **N / P ratio aboveground** | 54.7 < 0.001 | 47.0 | N > C > N+P > P | 35.7 < 0.001 | 25.8 |
| **N concentr. belowground** | 8.11 < 0.001 | 14.2 | N >  N+P & P & C | 20.4 < 0.001 | 30.5 |
| **P concentr. belowground** | 14.0 < 0.001 | 18.5 | N+P & P > C & N | 35.4 < 0.001 | 39.5 |
| **N / P ratio belowground** | 21.0 < 0.001 | 27.6 | N > C > N+P & P | 27.9 < 0.001 | 30.7 |

Background N availability was estimated at each site by comparing the N concentration in control plots with its average concentration in N and N+P plots and ranged from 0.495 to 0.952 (median = 0.813). Background P availability was estimated by comparing the P concentration in control plots with its average concentration in P and N+P plots and ranged from 0.625 to 0.917 (median = 0.730).

The change of aboveground biomass due to fertilisation also affected the amount of light available at the soil surface (F_3,25_=6.06, p = 0.003), with the largest decline (to 1.7% of the full radiation) observed in NP plots, followed by N plots, where light intensity declined to 2.7% of the full radiation, but was not significantly different from the other fertilisation treatments. Control plots received 10.5% of the full radiation at the soil surface.

## Summary of soil and biomass changes

Fig. EA3 provides a visual summary of the response of soil characteristics, biomass weight and concentration of nutrients in biomass to fertilisation treatment.


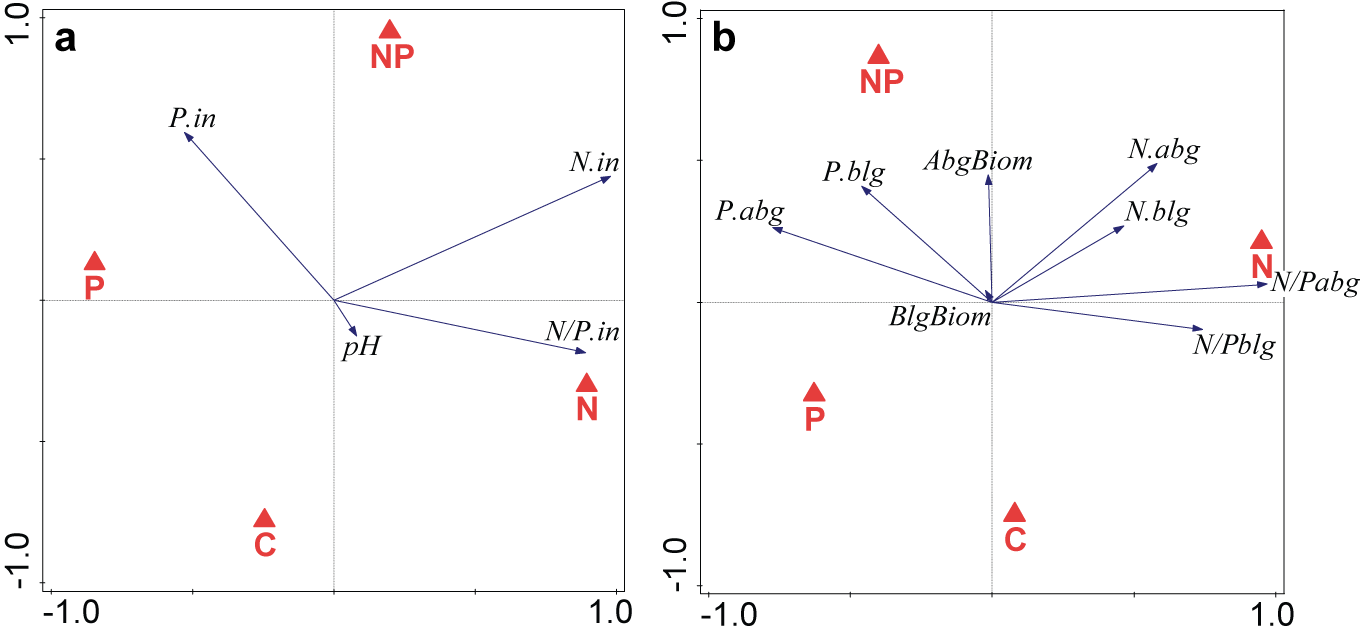


**Fig. EA3** – Statistical summary of the response of soil (**a**) and biomass (**b**) parameters to fertilisation treatment, using multivariate constrained ordination (RDA). The fertilisation treatment explains large fractions of the total variation in examined parameters (R^2^_adj_=16.4% for **a**, 21.6% for **b**). The fertilisation effects are shown by the triangle centroids that can be perpendicularly projected onto characteristics’ arrows to predict their average values. The arrow lengths reflect the relative proportion of explained variation and the cosine of the angle between a pair of characteristics approximates their linear correlation. All characteristics except pH were log-transformed and standardised to mean 0 and variance 1. *P.in* – concentration of P in soil in orthophosphate form, *N.in* – concentration of N in soil as nitrate and ammonium ions, *N/P.in* – N/P ratio of inorganic N and P forms; *P.*xxx – total P content in biomass, *N.*xxx – total N content in biomass, *N/P*xxx – the N/P ratio, xxx is either *abg* for aboveground or *blg* for belowground biomass. *AbgBiom* and *BlgBiom* represent, respectively, the dry weight of aboveground and belowground biomass.

## N and P in experimental seedlings

Content of macronutrients in the aboveground biomass of experimental seedlings is summarised in Table EA8, with respect to both the fertilisation treatment and plant taxonomic identity. Table EA8 also summarises the percentage colonisation of seedling roots by G-AMF and M-FRE.

**Table EA8** – Summary of fertilisation-based differences and the differences among host plant species in the content of nitrogen and phosphorus (both given as weight percentages), their ratio, and root length percentage colonisation by G‑AMF and M‑FRE, estimated in seedling roots by microscopy. Arithmetic averages are shown ± standard error, with the number of samples used in calculation given in parenthesis, The averages related to fertilisation treatment are computed from all species and the averages related to species identity use plants from all fertilisation treatments.

|  |  | **Nitrogen** | **Phosphorus** | **N/P ratio** | **AMF colonisation** | **FRE colonisation** |
| --- | --- | --- | --- | --- | --- | --- |
| **Fertilisation effects** | **Control plots** | 2.18 ± 0.10  (n=22) | 0.35 ± 0.02  (n=21) | 6.60 ± 0.34  (n=20) | 64.5 ± 2.8  (n=77) | 27.2 ± 2.2 (n=77) |
|  | **N plots** | 4.77 ± 0.28  (n=8) | 0.37 ± 0.03  (n=13) | 12.79 ± 1.25  (n=8) | 54.7 ± 4.8 (n=25) | 26.9 ± 5.1 (n=25) |
|  | **P plots** | 2.11 ± 0.07  (n=19) | 0.59 ± 0.02  (n=22) | 3.70 ± 0.15  (n=18) | 66.7 ± 2.7 (n=81) | 23.9 ± 1.8 (n=81) |
|  | **N+P plots** | 4.28 ± 0.33  (n=6) | 0.71 ± 0.03  (n=11) | 6.17 ± 0.34  (n=6) | 43.7 ± 4.7 (n=17) | 16.3 ± 5.0 (n=17) |
| **Host species effects** | **Plantago lanceolata** | 2.44 ± 0.33  (n=14) | 0.50 ± 0.05  (n=14) | 5.52 ± 0.90  (n=14) | 81.2 ± 2.2 (n=46) | 42.0 ± 2.7 (n=46) |
|  | **Centaurea jacea** | 2.14 ± 0.11  (n=11) | 0.44 ± 0.05  (n=14) | 5.41 ± 0.55  (n=11) | 80.9 ± 1.9 (n=35) | 23.7 ± 2.2 (n=35) |
|  | **Betonica officinalis** | 3.68 ± 0.55  (n=8) | 0.46 ± 0.07  (n=8) | 8.87 ± 1.70  (n=8) | 61.9 ± 4.2 (n=27) | 15.0 ± 2.0 (n=27) |
|  | **Anthoxanthum odoratum** | 3.11 ± 0.24  (n=19) | 0.51 ± 0.03  (n=21) | 6.85 ± 0.73  (n=19) | 52.7 ± 2.3 (n=65) | 25.4 ± 2.4 (n=65) |
|  | **Poa angustifolia** | 1.79 ± 0.08  (n=3) | 0.53 ± 0.06  (n=10) | 3.42 ± 0.09  (n=3) | 30.0 ± 3.9 (n=27) | 5.9 ± 1.8 (n=27) |

Table EA9 summarises the response of N and P concentration in seedling aboveground biomass (and their N/P ratio) to fertilisation. Seedlings from the same species and fertilisation treatment plots had to be pooled within a site and occasionally even from two neighbouring sites to approach the dry biomass weight required for nutrient quantification. Fertilisation with N or N+P increased N concentration, fertilisation with P or N+P increased P concentration. The N/P ratio was increased by adding only N, decreased by adding only P, and it was not changed by N+P treatment compared to control plots.

**Table EA9** – Analyses of the response of N and P concentration and their N/P ratio in seedling aboveground biomass to fertilisation treatment and site identity. The degrees of freedom for F‑statistics vary across response variables (due to missing values), all χ^2^-statistics have 1 degree of freedom.

|  | **Treatment effect** | | | **Among-site variation** | |
| --- | --- | --- | --- | --- | --- |
|  | **Test (DF) F / p** | **Explained variation [%]** | **Effect description** | **Test χ^2^ p** | **Explained variation** |
| **N concentration** | (3, 42) 85.8 / < 0.001 | 51.9 | N & N+P > P & C | 17.6  < 0.001 | 12.6 |
| **P concentration** | (3, 54) 52.9 / < 0.001 | 55.8 | N+P > P > N & C | 16.7 < 0.001 | 19.0 |
| **N / P ratio** | (3,40) 86.0 / < 0.001 | 65.8 | N > N+P & C > P | 13.1 < 0.001 | 9.6 |

## References

Bartoň K (2024) MuMIn: multi-model inference. R package version 1.48.4, URL: https://cran.r-project.org/package=MuMIn.

Hothorn T, Bretz F, Westfall P (2008) Simultaneous inference in general parametric models. Biometrical Journal 50(3): 346–363.

Legendre P, Legendre L (2012) Numerical Ecology. 3rd English edition. Amsterdam, 950 pp., Elsevier, Amsterdam, The Netherlands.

Pinheiro J, Bates D, R Core Team (2023) nlme: linear and nonlinear mixed effects models. R package version 3.1-164, URL: https://cran.r-project.org/package=nlme.

# Appendix EA7 – Interaction between nutrients addition and host plant identity

This section evaluates how the fertilisation effects varied among five host plant species. We found no significant interaction of P addition with host identity for any of the examined response variables, but there were multiple response variables with a significant effect of interaction between N addition and host species identity, with most of them concerning G‑AMF symbionts (Table EA10). Strongest response was found in the G‑AMF community variation, summarised in Fig. EA4. The two subplots summarise together the pattern in a subspace of the first three constrained axes that all had a significant marginal effect. The first axis (horizontal axis in both subplots) summarises the compositional differences between forb and grass host species, with the community in grass roots characterised by a higher relative abundance of three Archaeosporaceae VTXs. The second axis (vertical axis of Fig. EA4a) characterises changes due to N addition together with the third axis (vertical axis of Fig. EA4b), which shows the compositional changes in the G‑AMF community of the *Poa angustifolia* roots, shared with the other host species. Less varying positions of the N+ centroids along the first constrained axis, as compared with the N- centroids, represents the convergence of G‑AMF community composition after N addition. It is also apparent that the arrows connecting centroids with and without N addition for a particular host species are shorter for grass species, representing smaller change in the composition of G‑AMF community due to N addition.

G-AMF community also responded to N addition by decreasing its taxonomic richness and α‑diversity, but only in the forb hosts (Table EA10). This matches the larger compositional changes due to N addition in Fig. EA4.

**Table EA10** – The summary of statistical models examining the interaction effect of N or P addition with plant host species identity on the composition, α‑diversity (Hill’s N2 index), taxonomic richness, colonisation level, and G‑AMF/M‑FRE ratio of DNAs of G-AMF and M-FRE communities, using partial RDA (for compositional variation) and LMM (for other community attributes). Test statistic for RDA is pseudo-F (used without DFs) and likelihood-ratio (χ^2^) statistic for LMM, with DF=4. AO – *Anthoxanthum odoratum*, BO – *Betonica officinalis*, CJ – *Centaurea jacea*, PA – *Poa angustifolia*, PL – *Plantago lanceolata*. *n. s.* – not significant, *n. a.* – not appropriate. ▲ is an increase with N addition, ▼ a decrease with N addition, × no change. Significance estimates with 0.05 < p < 0.10 are shown, but in parenthesis and they are interpreted as a non-significant outcome of the test.

|  | **N addition : host species interaction** | | | **P addition : host species interaction** | | |
| --- | --- | --- | --- | --- | --- | --- |
|  | **Test statistic p-value** | **Explained variation [%]** | **Effect description** | **Test statistic p-value** | **Explained variation [%]** | **Effect description** |
| **Variation of G-AMF community** | F = 1.6 p < 0.001 | 0.6% | Fig. EA4 | F = 1.0 n. s. | < 0.1% | n. a. |
| **Variation of M-FRE community** | F = 0.9 n. s. | < 0.1% | n. a. | F = 0.7 n. s. | < 0.1% | n. a. |
| **α-diversity of G-AMF community** | χ^2^ = 11.2 p = 0.025 | 3.1% | ▼ in forb species only | χ^2^ = 5.6 n. s. | 1.5% | n. a. |
| **Richness of G-AMF community** | χ^2^ = 11.9 p = 0.018 | 3.2% | ▼ in forb species only | χ^2^ = 4.1 n. s. | 1.1% | n. a. |
| **α-diversity of M-FRE community** | χ^2^ = 3.6 n. s. | 0.9% | n. a. | χ^2^ = 9.2 n. s. (p = 0.055) | 2.4% | n. a. |
| **Richness of M-FRE community** | χ^2^ = 10.1 p = 0.040 | 2.2% | BO no ▼,  PA slight ▲ | χ^2^ = 1.9 n. s. | 0.4% | n. a. |
| **G-AMF/M-FRE ratio** | χ^2^ = 2.9 n. s. | 0.6% | n. a. | χ^2^ = 0.8 n. s. | 0.1% | n. a. |
| **G-AMF root  colonisation** | χ^2^ = 11.1 p = 0.025 | 1.4% | PA ▲, BO ▼, AO, CJ, PL × | χ^2^ = 3.6 n. s. | 0.7% | n. a. |
| **M-FRE root colonisation** | χ^2^ = 9.1 n. s. (p = 0.058) | < 0.1% | n. a. | χ^2^ = 3.2 n. s. | 0.5% | n. a. |


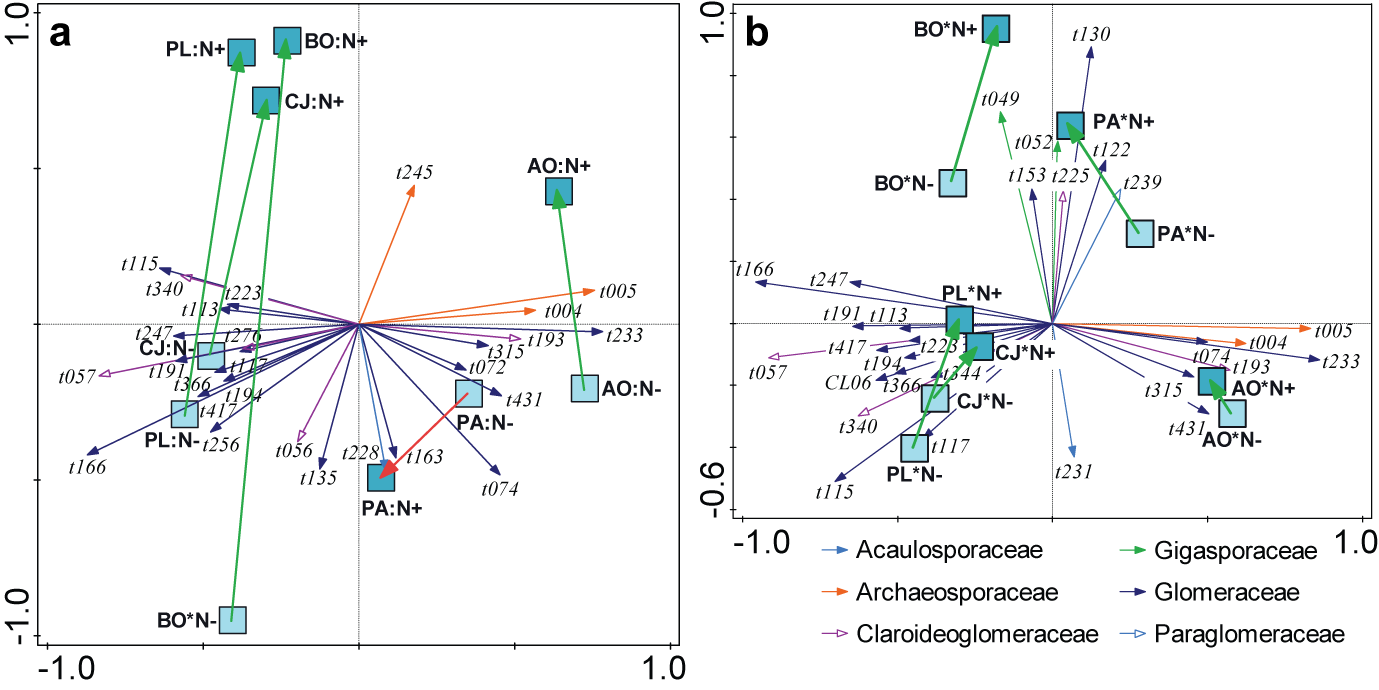


**Fig. EA4** – The effects of N addition (N+ vs N-), host plant species (AO – *Anthoxanthum odoratum*, BO – *Betonica officinalis*, CJ – *Centaurea jacea*, PA – *Poa angustifolia*, PL – *Plantago lanceolata*), and their interaction on the G-AMF community composition, shown as biplots using the first two constrained axes (**a**) and the first and third constrained axis (**b**). The first to third axis explains (R^2^_adj_=) 3.3%, 1.2%, and 0.8% of the total community variation, respectively. The arrow attributes show the assignment of VTXs to G‑AMF families (see the key in lower right corner). Average relative abundance of individual VTXs for different combinations of host species (SP) and N addition or N absence can be interpreted by perpendicularly projecting SP:N+ or SP:N- centroids onto (prolonged) VTX arrows. Green arrows (plus red arrow for PA in plot **a**) connect two centroids of a particular host species for N- and N+ plots. N- plots also include P-only plots, and N+ plots include N+P plots.
